# Recent Development of Nickel-Based Electrocatalysts for Urea Electrolysis in Alkaline Solution

**DOI:** 10.3390/nano12172970

**Published:** 2022-08-27

**Authors:** Krishnan Shanmugam Anuratha, Mia Rinawati, Tzu-Ho Wu, Min-Hsin Yeh, Jeng-Yu Lin

**Affiliations:** 1 Department of Chemical and Materials Engineering, Tunghai University, Taichung City 40704, Taiwan; 2 Department of Chemical Engineering, National Taiwan University of Science and Technology, Taipei 10607, Taiwan; 3 Department of Chemical and Materials Engineering, National Yunlin University of Science and Technology, Yunlin 64002, Taiwan

**Keywords:** urea electrolysis, nickel, electrocatalysts, alkaline medium

## Abstract

Recently, urea electrolysis has been regarded as an up-and-coming pathway for the sustainability of hydrogen fuel production according to its far lower theoretical and thermodynamic electrolytic cell potential (0.37 V) compared to water electrolysis (1.23 V) and rectification of urea-rich wastewater pollution. The new era of the “hydrogen energy economy” involving urea electrolysis can efficiently promote the development of a low-carbon future. In recent decades, numerous inexpensive and fruitful nickel-based materials (metallic Ni, Ni-alloys, oxides/hydroxides, chalcogenides, nitrides and phosphides) have been explored as potential energy saving monofunctional and bifunctional electrocatalysts for urea electrolysis in alkaline solution. In this review, we start with a discussion about the basics and fundamentals of urea electrolysis, including the urea oxidation reaction (UOR) and the hydrogen evolution reaction (HER), and then discuss the strategies for designing electrocatalysts for the UOR, HER and both reactions (bifunctional). Next, the catalytic performance, mechanisms and factors including morphology, composition and electrode/electrolyte kinetics for the ameliorated and diminished activity of the various aforementioned nickel-based electrocatalysts for urea electrolysis, including monofunctional (UOR or HER) and bifunctional (UOR and HER) types, are summarized. Lastly, the features of persisting challenges, future prospects and expectations of unravelling the bifunctional electrocatalysts for urea-based energy conversion technologies, including urea electrolysis, urea fuel cells and photoelectrochemical urea splitting, are illuminated.

## 1. Background

Due to the overall rising need for energy, the depletion of fossil fuels, concerns about pollution and global warming issues, a great deal of attention has recently been given to clean and renewable energy [1,2]. As an alternative, hydrogen (H_2_) is recognized as a potential carbon-free energy carrier since it has the features of high gravimetric energy density, eco-friendly nature, abundance and produces no emissions (water is the only product). As of now, the most sustainable technique for H_2_ generation is electrocatalytic water electrolysis—a clean and secure technology due to plentiful water resources and high-purity products. Generally, a water-splitting reaction is made up of two half-reactions: the cathodic hydrogen evolution reaction (HER) and the anodic oxygen evolution reaction (OER). The thermodynamic voltage for water electrolysis is 1.23 V; however, the sluggish kinetics of anodic OER are due to the multiproton-coupled electron transfer processes which have a high activation energy barrier for O-O bond formation, thereby resulting in the high operating voltage of above 1.8 V [3]. This constraint can be addressed by substituting the difficult OER with other more easily oxidized molecules, such as hydrazine, alcohols, amine, aldehyde and urea, since they offer promising energy-saving H_2_ generation [4,5,6]. Among the aforementioned alternative oxidized molecules, the urea oxidation reaction (UOR) has recently received attention, as an energy-saving urea electrolysis approach that allows for simultaneous H_2_ generation and urea-rich wastewater cleansing, which holds great significance on energy storage and global environmental problems [7]. Urea is a key hydrogen carrier, and urea splitting has a low theoretical decomposition potential of 0.37 V, compared to H_2_O electrolysis (1.23 V) for H_2_ generation. Some excellent characteristics of urea molecules (CO(NH_2_)_2_) are abundance, low-cost (as it can be obtained from industry production and human/animal urine) and 36% lower cost and 30% less energy required for H_2_ generation when compared to traditional H_2_O electrolysis. Thereby, urea could be considered as the best raw material for fuel cells [3,8].

**Figure 1:**
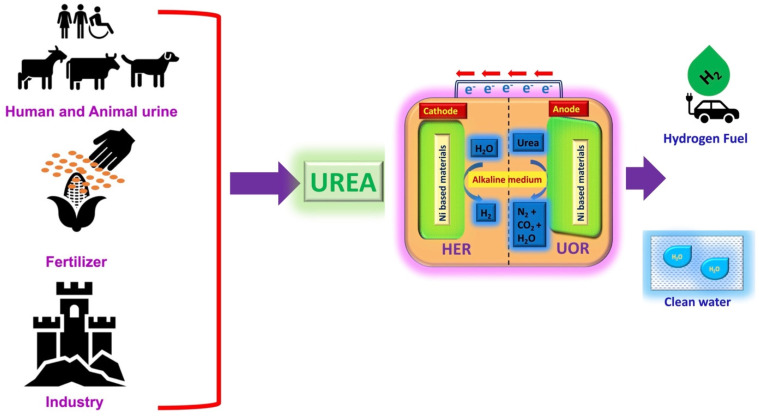
Schematic illustration of urea electrolysis for H_2_ generation and the sources of urea.

**Figure 2:**
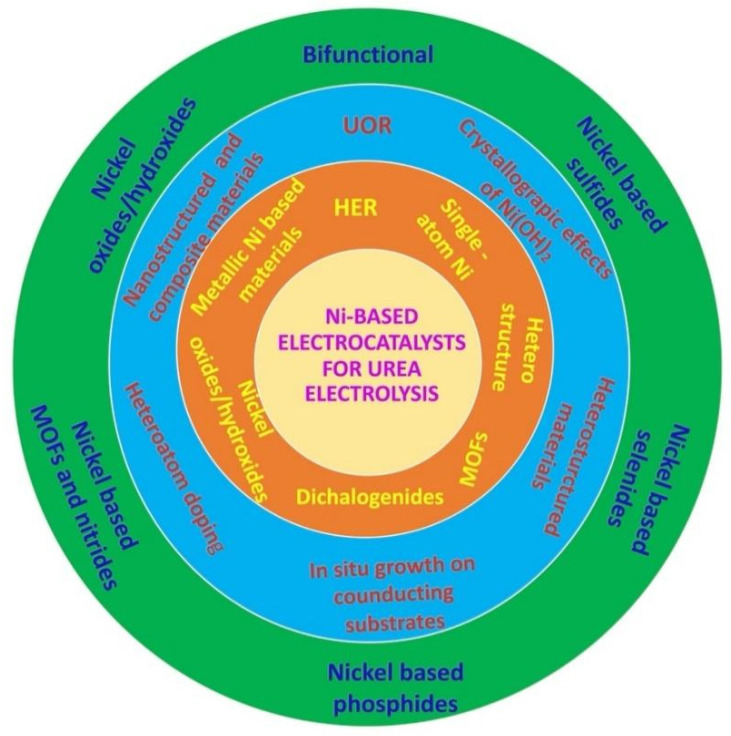
Scheme organization of the Ni-based HER, UOR and the bifunctional electrocatalysts discussed in this review.

Typically, urea electrolysis involves anodic UOR and cathodic HER in alkaline medium, and their fundamentals are explained in the following sections. Figure 1 shows the schematic representation of urea sources and urea electrolysis for H_2_ production and its application. Commonly, active electrocatalysts are utilized to overcome the activation barriers and sluggish kinetics of the UOR and HER, with these electrocatalysts lowering overpotentials and accelerating the reaction rate [9]. Noble platinum (Pt) was the earliest commercial electrocatalyst for both the HER and UOR [3,10]. Recently, nickel-based materials have been regarded as highly efficient and non-precious electrocatalysts for both the HER and UOR since they reduce costs and speed up reaction kinetics [3,11]. Most of the HER electrocatalysts are exploited monofunctionally and display low activity for the UOR and vice versa. Those inadequacies can be conquered by the emerging bifunctional UOR/HER electrocatalysts. Therefore, this review article concentrates on recent advances related to monofunctional/bifunctional nickel-based electrocatalysts for both the HER and UOR in alkaline medium to develop energy-saving pathways through urea electrolysis for H_2_ generation. Figure 2 demonstrates the overall organization of the Ni-based HER, UOR and the bifunctional electrocatalysts explained in this review article. Finally, challenges and future perspectives on scientific issues related to improving the field of urea electrolysis are also highlighted in this review article.

Initially, the extraction of H_2_ and N_2_ from urine or alkaline solution comprised of urea at 1.5 V potential using Hoffmann apparatus was performed by Boggs et al. in 2009 [12]. Figure 1 illustrates the operating principle of urea electrolysis in alkaline medium. As said before, urea electrolysis comprises two half-cell reactions: For the anodic UOR, the addition of 1 mole urea and 6 moles hydroxyl ions (OH^−^) generally results in products of N_2_, H_2_O and CO_2_, as depicted in Equation (1). The cathodic HER produces H_2_ through the reduction of H_2_O (Equation (2)) and, subsequently, the overall reactions of urea electrolysis (UOR + HER) generate H_2_, N_2_ and CO_2_ (Equation (3)) in alkaline medium. Both anodic and cathodic reactions belong to the six electron transfer process and the theoretical thermodynamic potentials of Equations (1)–(3) are −0.46 V, −0.83 V and 0.37 V, respectively, with their corresponding reactions shown as follows [13]: 

Anode (UOR) in alkaline media (pH ~ 14):

(1) $$\mathrm{CO}{\left({\mathrm{NH}}_{2}\right)}_{2}+6{\mathrm{OH}}^{-}\to N_{2}+5H_{2}O+{\mathrm{CO}}_{2}+6e^{-}\;E^{o}=-0.46\;V\;\mathrm{vs}.\;\mathrm{SHE}$$

Cathode (HER) in alkaline media (pH ~ 14):

(2) $$6H_{2}O+6e^{-}\to 3H_{2}+6{\mathrm{OH}}^{-}\;E^{o}=-0.83\;V\;\mathrm{vs}.\;\mathrm{SHE}$$

Overall catalytic reaction (UOR/HER):

(3) $$\mathrm{CO}{\left({\mathrm{NH}}_{2}\right)}_{2}+H_{2}O\to N_{2}+3H_{2}+{\mathrm{CO}}_{2}\;E^{o}=0.37\;V$$

## 2. Ni-Based Electrocatalysts for UOR Application

### 2.1. UOR Catalytic Mechanisms in Alkaline Medium

Nevertheless, the UOR involves six electrons in the reaction process; therefore, it usually requires high overpotentials which hinder the overall kinetics of urea electrolysis [3]. As such, the quest for advancing UOR electrocatalysts is highly desirable but still challenging. Therefore, rational design of catalysts for efficient electro-oxidation of urea is of great importance in this booming research field. Although the required theoretical thermodynamic potential for generating H_2_ from urea electrolysis is 0.37 V, it still needs high potential to conduct urea electrolysis practically. The utilization of low-cost and non-precious nickel (Ni)-based electrocatalysts shows promising UOR catalytic activity in alkaline solution among the various noble and high-cost catalysts (Pt, Pt−Ir, Rh), as revealed by the pioneering work reported by Botte’s research group [12]. Since then, the electrochemical performances of diverse Ni-based catalysts have been investigated in alkaline UOR application. Understanding the underlying mechanisms is crucial to further develop the advanced catalysts towards the UOR. Under alkaline conditions, metallic nickel spontaneously transforms into Ni(OH)_2_ on the electrode surface. When oxidative potentials are applied on the anode, Ni(OH)_2_ can be oxidized to NiOOH. As such, early mechanistic studies mainly focus on the NiOOH/Ni(OH)_2_ redox couple in UOR catalytic application, revealing direct and indirect mechanisms [12,14,15,16]. Very recently, the reaction mechanism of a different category of Ni-based electrocatalyst, nickel ferrocyanide Ni_2_Fe(CN)_6_, was investigated [17], demonstrating a two-stage reaction pathway involving an ammonia intermediate. Therefore, in this section, we summarized three proposed catalytic mechanisms of Ni-based electrocatalysts in alkaline UOR application. They are known as direct and indirect oxidation mechanisms for the NiOOH/Ni(OH)_2_ catalyst and a two-stage mechanism for the Ni_2_Fe(CN)_6_ catalyst. In addition, two main design principles for obtaining high-performance UOR catalysts are discussed in this section, including activating more active sites and enhancing intrinsic UOR catalytic activity towards urea oxidation.

#### 2.1.1. Direct Oxidation Mechanism for the NiOOH/Ni(OH)_2_ Catalyst

Direct mechanism describes the adsorption of urea molecules on Ni^3+^ active sites, and the adsorbed urea reacts with OH^−^ from the alkaline electrolyte giving CO_2_ and N_2_ [12]. Although the UOR can be described as a single chemical equation (Equation (1)), the decomposition of urea involves multiple reaction steps in the process. Assisted by density functional theory (DFT), three possible pathways for urea electro-oxidation in alkaline media were evaluated [14]. In this work, the rate constants and free energies for each intermediate step were calculated. The results indicated that the adsorption of urea molecules is the prerequisite for all pathways. Moreover, a bridge-coordinated structure was found to be the energetically favorable form, having the N or O atom of urea connected to the Ni^3+^ active sites on the electrode surface and the O atom of urease interacting with the C atom of urea. After urea decomposition, the adsorbed CO_2_ on Ni^3+^ active sites could further react with OH^−^ (either from the alkaline electrolyte or the adsorbed OH^−^ on adjacent NiOOH) to regenerate NiOOH active sites. Notably, the rate constant corresponding to CO_2_ desorption is greatly lower than other steps [14]. Thus, removing the produced CO_2_ from the catalyst surface is regarded as the rate-determining step for the UOR. The chemical equations for the direct mechanism are defined as the following: 

Electrochemical reaction:

(4) $$6\mathrm{Ni}{\left(\mathrm{OH}\right)}_{2}+6{\mathrm{OH}}^{-}⇌6\mathrm{NiOOH}+6H_{2}O+6e^{-}$$

NiOOH catalytic reaction:

(5) $$\mathrm{CO}{\left({\mathrm{NH}}_{2}\right)}_{2}+6{\mathrm{OH}}^{-}\;\overset{\mathrm{NiOOH}}{\to }{\;N}_{2}+{\mathrm{CO}}_{2}+5H_{2}O+6e^{-}$$

In this regard, Ni(OH)_2_ can be considered a pre-catalyst [18]. In order to exhibit UOR catalytic activity, Ni(OH)_2_ has to be electrochemically oxidized to NiOOH with Ni^3+^ active sites (Equation (4)). The active NiOOH catalyzes the decomposition of urea without reverting to Ni(OH)_2_ (Equation (5)).

#### 2.1.2. Indirect Oxidation Mechanism for the NiOOH/Ni(OH)_2_ Catalyst

**Figure 3:**
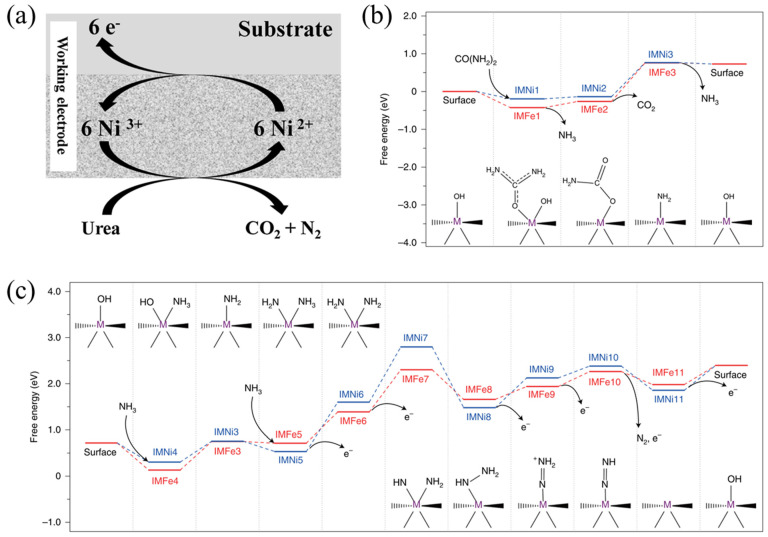
(**a**) Illustration of the indirect oxidation mechanism for the Ni(OH)_2_ catalyst and two-stage reaction mechanism diagrams for the Ni_2_Fe(CN)_6_ catalyst in (**b**) the first stage (the reaction from urea to NH_3_) and (**c**) the second stage (the reaction from NH_3_ to N_2_). (**a**) Reprinted with permission from Ref。 [15]. (**b**,**c**) Reprinted with permission from Ref. [17].

An indirect mechanism was proposed based on the observations from in situ Raman microscopy [15] and X-ray diffraction [16]. In these two works, the elaborate design of experiments conducted on various electrolytes (pure KOH, pure urea and KOH with urea) allowed for elucidation of the reaction mechanism of the UOR. In this mechanism, Ni(OH)_2_ acts as the electrocatalyst for the UOR. Ni(OH)_2_ first undergoes an electrochemical reaction to obtain NiOOH at oxidative potentials (Equation (4)). Ni^3+^ serves as the active site to react with a urea molecule in a chemical reaction (Equation (6)). In this reaction, urea can be decomposed into CO_2_ and N_2_, while NiOOH can be chemically reduced to regenerate the Ni(OH)_2_ catalyst. Upon applying oxidative potentials, the combination of electrochemical and chemical reactions can be maintained for electro-oxidation of urea. An illustration of the indirect mechanism for the Ni(OH)_2_ catalyst is shown in Figure 3a, and the reactions can be expressed as the following:

Electrochemical reaction:

(4) $$6\mathrm{Ni}{\left(\mathrm{OH}\right)}_{2}+6{\mathrm{OH}}^{-}⇌6\mathrm{NiOOH}+6H_{2}O+6e^{-}$$

NiOOH chemical reaction:

(6) $$6\mathrm{NiOOH}+\mathrm{CO}{\left({\mathrm{NH}}_{2}\right)}_{2}+H_{2}O\to 6\mathrm{Ni}{\left(\mathrm{OH}\right)}_{2}+N_{2}+{\mathrm{CO}}_{2}$$

The oxidation of Ni(OH)_2_ to NiOOH at the anode (Equation (2)) is a competing reaction since it attributes to current during electrolysis and occurs at 0.49 V vs. SHE. Therefore, the applied potential is determined by the Ni-redox potentials while using the Ni-redox-based electrocatalysts, not the oxidation potential of urea [12,14,15]. Moreover, Schechter et al. also employed in situ Raman spectroscopic measurements to examine the reaction mechanism of an Ni/Sn electrode in UOR application [19]. The results confirm the formation of NiOOH on the electrode surface, and electro-oxidation of urea is initiated by Ni^3+^OOH species. In addition, Peng and coworkers revealed that high-valent Ni^4+^ active sites exhibit higher UOR activity in comparison to Ni^3+^ [4]. In this work, the authors utilized in situ Fourier transform infrared spectroscopy (FTIR) coupled with DFT calculations to propose a lattice-oxygen-involved reaction pathway for Ni^4+^. The results indicated that the CO_2_ desorption energy barrier can be significantly reduced, and hence boost overall UOR performance. Notably, the direct and indirect oxidation mechanisms could simultaneously take place in UOR application. Cao et al. systematically investigated several key parameters, such as polarization potential and KOH concentration, by conducting electrochemical impedance spectroscopy (EIS) analyses [20]. An equivalent circuit model was proposed to evaluate the resistances associated with the direct and indirect pathways during UOR operation.

#### 2.1.3. Two-Stage Mechanism for the Ni_2_Fe(CN)_6_ Catalyst

As mentioned earlier, direct and indirect oxidation mechanisms are recognized as the prevailing mechanisms for NiOOH/Ni(OH)_2_. Both mechanisms describe the redox transition between Ni^3+^ and Ni^2+^ in the UOR catalytic process. In 2021, Qiao et al. [17] revealed a distinctive reaction mechanism for nickel ferrocyanide (Ni_2_Fe(CN)_6_. The Ni_2_Fe(CN)_6_ belongs to Prussian blue analogues, which are also capable of undergoing Ni^3+^/Ni^2+^ redox transition in alkaline solution. However, Ni_2_Fe(CN)_6_ was shown to maintain at Ni^2+^ and Fe^2+^ throughout the UOR tests, as evidenced by in situ X-ray absorption spectroscopic analyses. NiOOH species cannot be detected through in situ Raman and synchrotron radiation Fourier transform infrared examinations, indicating the UOR’s catalytic reaction is not realized by Ni^3+^ active sites. This behavior is different from the direct and indirect oxidation mechanisms for NiOOH/Ni(OH)_2_. Combined with DFT calculations, the reaction mechanism diagrams for the Ni_2_Fe(CN)_6_ catalyst in the UOR were proposed (Figure 3b,c). The chemical equations were described as the following:

(7) $$\mathrm{CO}{\left({\mathrm{NH}}_{2}\right)}_{2}+H_{2}O\to {\mathrm{CO}}_{2}+2{\mathrm{NH}}_{3}$$

(8) $$2{\mathrm{NH}}_{3}+6{\mathrm{OH}}^{-}\to N_{2}+6H_{2}O+6e^{-}$$

where Equation (7) takes place near Ni^2+^ active sites under alkaline condition producing CO_2_ and NH_3_. The intermediate NH_3_ can be further converted to N_2_ and H_2_O on Fe^2+^ sites on the electrode surface (Equation (8)). Of note, this two-stage reaction mechanism also involves chemical and electrochemical reactions in the UOR process. Nevertheless, the reaction pathway is drastically different from the previously understood direct and indirect mechanisms. 

### 2.2. Strategies for Developing Advanced UOR Electrocatalysts

As discussed in previous section, the UOR catalytic process is recognized as a complex reaction involving multiple reaction steps. Therefore, developing advanced UOR electrocatalysts that can enable efficient electro-oxidation of urea is in great demand. Since the UOR has received increasing attention in recent years, several reviews have summarized the progression and achievements related to the UOR [18,21,22,23]. In addition, Cao et al. [24] compared the electrochemical performances of various Ni-based catalysts in the UOR and their applications in direct urea fuel cells (DUFCs). Zou et al. [25] summarized recent progress not only related to electro-oxidation of urea, but also for photoelectrochemical urea splitting. Abdelkareem et al. [26] reviewed the catalytic performances of metal chalcogenides used in DUFCs. With the efforts of these reviews, UOR performance for diverse catalysts can be compared, and the practical applications of these electrocatalysts in urea electrolysis, DUFCs and photoelectrochemical urea splitting could be evaluated. However, these reviews mainly sorted by the correlation between crystal structures/chemical compositions of catalysts and their catalytic performances. The current review focuses on design principles, which are rarely reported. An ideal UOR electrocatalyst renders boosted catalytic current responses in urea-containing electrolytes with reduced overpotentials. In other words, it is highly desirable to obtain advanced catalysts allowing high current densities and low applied potentials in UOR application. In this review, we summarized two main design principles for electrocatalysts with boosted UOR performance, i.e., activating more active sites and enhancing intrinsic catalytic activity towards urea oxidation.

#### 2.2.1. Activating More Active Sites for the UOR

##### Nanostructured and Composite Materials

**Figure 4:**
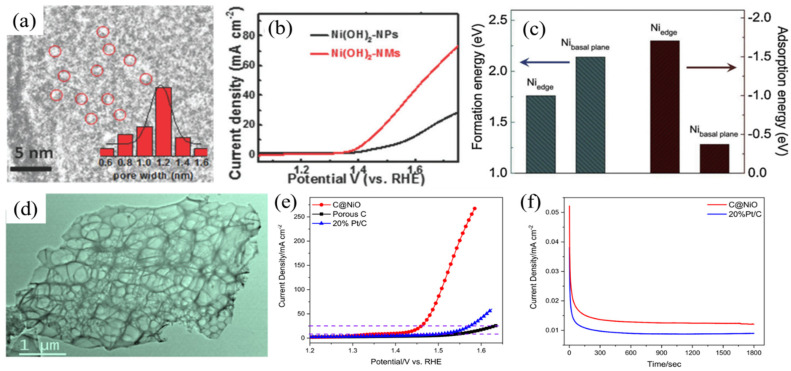
(**a**) TEM image of Ni(OH)_2_-NMs; (**b**) comparison of LSV curves of Ni(OH)_2_-NMs and Ni(OH)_2_-NPs in 1 M KOH with 0.33 M urea; (**c**) the calculated formation energies of NiOOH from Ni(OH)_2_ and adsorption energies of urea on the edge and basal plane of Ni(OH)_2_; (**d**) TEM image of C@NiO; comparison of (**e**) LSV curves and (**f**) chronoamperometric curves of C@NiO and commercial Pt/C in 1 M KOH with 0.33 M urea. (**a**,**b**) Reprinted with permission from Ref. [28]. (**c**) Reprinted with permission from Ref. [30]. (**d**–**f**) Reprinted with permission from Ref. [32].

As with many electrochemical applications, electrochemically active surface areas (ECSAs) hold the key to improved electrochemical performance. In the UOR, providing more exposed active sites is usually beneficial for achieving higher catalytic current. Botte et al. [27] used a surfactant-assisted method which successfully prepared exfoliated Ni(OH)_2_ nanosheets (with a thickness of ~1 nm). This sample exhibits high current density of 154 mA cm^–2^ mg^–1^ at 1.42 V (vs. reversible hydrogen electrode, RHE), which is 170 times higher than that of its bulk Ni(OH)_2_ counterpart. Li and coworkers demonstrated that atomically thick Ni(OH)_2_ nanomesh (denoted as Ni(OH)_2_–NM, Figure 4a) can be obtained from NiCl_2_–K_2_Ni(CN)_4_ cyanogel, while the use of NiCl_2_ precursor results in aggregated and irregular Ni(OH)_2_ particles (denoted as Ni(OH)_2_–NPs) [28]. Based on the double-layer capacitance evaluations, the ECSA of Ni(OH)_2_–NM electrodes is 111.43 m^2^ g^–1^, which is much higher than that of Ni(OH)_2_–NPs (43.07 m^2^ g^–1^). Profiting from high ECSA and abundant nanoholes (~1.2 nm diameter), Ni(OH)_2_–NM catalyst exhibits enhanced UOR performance in comparison with Ni(OH)_2_–NPs (Figure 4b). In addition to ultrathin Ni(OH)_2_, Ye et al. [29] directly grew Ni(OH)_2_ onto nickel foam (NF) substrate. By changing reaction temperatures, different surface morphologies of Ni(OH)_2_ were obtained, including nanosheets and sheet-like, flower-like and twine-like surfaces. From their results, nanosheet morphology exhibits the highest catalytic activity for UOR application, with current density reaching 337 mA cm^–2^ at 1.48 V (vs. RHE). Recently, Luo et al. [30] revealed that the edges of Ni(OH)_2_ exhibit higher activity not only for forming Ni^3+^ species (NiOOH), but also the adsorption of urea molecules compared to the basal planes (Figure 4c), as evidenced by experimental results and DFT calculations. This is also the reason for the high mass activity of the Ni(OH)_2_-NMs catalyst, which has an abundance of exposed edges enabled by rich nanoholes, as observed in Li’s work [28]. 

Incorporating conductive polymer and/or carbon-based materials has been found to be an effective way to improve overall electrical conductivity for electrocatalysts. Song et al. [31] prepared polypyrrole/graphene oxide (PPy/GO) composite material and then immersed it in a Ni^2+^-containing solution. Guided by the interaction between Ni^2+^ and -NH-functional groups in PPy chains, nanostructured Ni(OH)_2_ can be obtained via a chemical precipitation method. The obtained Ni(OH)_2_/PPy/GO composite has good conductivity and favorable porosity, showing its potential for UOR application. Liu et al. [32] synthesized NiO nanoparticles anchored on highly porous carbon (C@NiO, Figure 4d) derived from eggshell membranes. Benefitting from high porosity and conductivity, the C@NiO catalyst exhibits ca. 200 mA cm^–2^ at 1.55 V (vs. RHE), which is superior to the commercial Pt/C catalyst (Figure 4e,f). Nonetheless, it is to be noted that carbon is thermodynamically unstable at high potentials in aqueous solution, from which the oxidation of carbon could result in severe carbon corrosion [33]. In such cases, the loss of highly conductive carbon support undermines catalytic stability in long-term UOR tests. Apart from the catalysts with high Ni content, embedding active nickel ions (Ni^3+^) in nanostructured MnO_2_ has also been reported as a promising candidate [34]. Notably, leveraging nanostructured materials and integrating Ni-based catalysts with highly conductive materials guarantees rich and exposed edge sites, which are crucial for catalytic reaction. 

##### In Situ Growth on Highly Conductive Substrates

**Figure 5:**
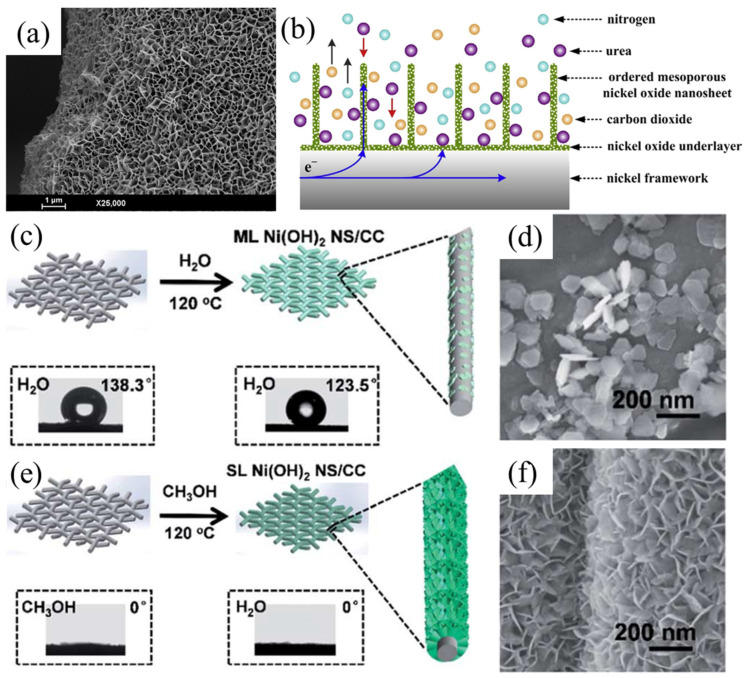
(**a**) SEM image and (**b**) illustration of vertically aligned NiO nanosheets in UOR application; (**c**,**e**) illustration of the synthetic process and (**d**,**f**) SEM image of (**c**,**d**) multilayer Ni(OH)_2_ in water and (**e**,**f**) single layer Ni(OH)_2_ in methanol solution. (**a**,**b**) Reprinted with permission from Ref. [35]. (**c**–**f**) Reprinted with permission from Ref. [37].

In situ growth of active materials on highly conductive substrates has also been demonstrated as an efficient approach. Wu et al. [35] demonstrated that vertically aligned NiO nanosheets can be directly grown onto NF substrate (Figure 5a). The large open structure between vertically aligned nanosheets allows fast transport of electrolyte, urea and gases, and the highly conductive NF substrate facilitates electron conduction (Figure 5b). As a result, high catalytic current density of 330 mA cm^–2^ mg^–1^ can be achieved at 1.59 V (vs. RHE). Wang et al. [36] revealed that using NH_4_F additive can tune the height/thickness of NiO nanowalls on NF substrate. Assisted by NH_4_F, the tailored NiO nanowall catalyst exhibits improved UOR performance. Moreover, its morphology can be retained after 12-h UOR tests, while the one without NH_4_F additive collapsed. Jin et al. [37] demonstrated that the solvent species could play a crucial role in growing Ni(OH)_2_ onto conductive carbon cloth (CC) substrate. The use of methanol greatly improves electrode/electrolyte compatibility, which helps the uniform growth of Ni(OH)_2_ on CC (Figure 5c–f). Moreover, the water-deficient environment would reduce the growth rate of Ni(OH)_2_, which is favorable for the formation of ultrathin nanosheet morphology with a thickness of ~0.8 nm. Thus, the prepared catalyst demonstrates promising catalytic performance with 436.4 mA cm^–2^ at 1.53 V (vs. RHE). These results indicate that in situ-grown Ni-based catalysts on highly conductive substrates forming nanoarrays with favorable morphologies can enhance overall electrical conductivity of electrodes and facilitate mass transports of urea and products, thus greatly enhancing UOR performance.

##### Heterostructured Materials

**Figure 6:**
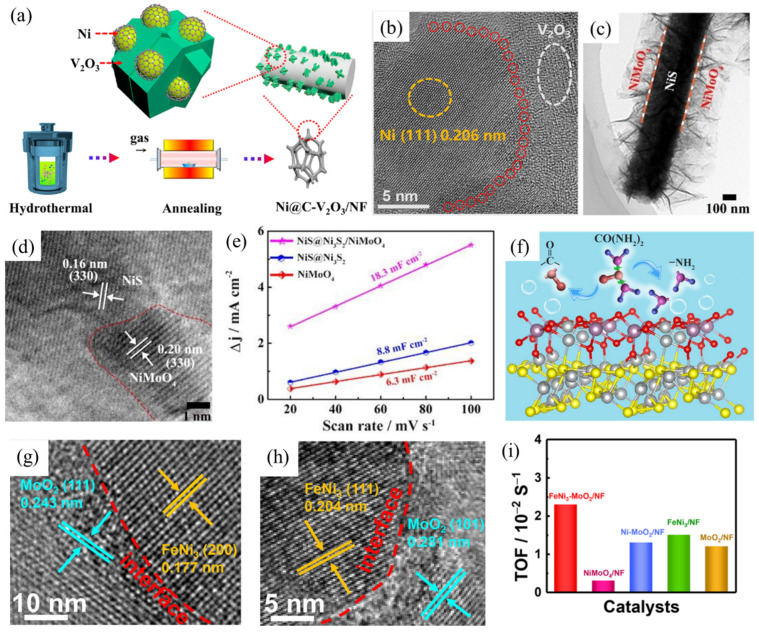
(**a**) Illustration of the preparations undertaken and (**b**) TEM image of Ni@C-V_2_O_3_/NF; (**c**,**d**) TEM images of NiS@Ni_3_S_2_/NiMoO_4_; (**e**) evaluation of C_dl_ values for NiS@Ni_3_S_2_/NiMoO_4_ and controlled samples; (**f**) the proposed UOR catalytic mechanism of NiS@Ni_3_S_2_/NiMoO_4_; (**g**) TEM images and (**i**) TOF values of FeNi_3_-MoO_2_ and controlled samples. (**a**,**b**,**g**–**i**) Reprinted with permission from Refs. [41,45]. (**c**–**f**) Reprinted with permission from Ref. [42].

Recently, rational design of heterogeneous nanostructures has been demonstrated as a promising approach to create rich active sites, provide electron-reconfigured interfaces and improve mass transport [38,39], which leads to enhanced UOR performance. MacFarlane et al. [40] fabricated MnCo_2_O_4_ nanoflakes onto NF substrate by hydrothermal reaction followed by electrodeposition of MnO_2_. The prepared MnO_2_/MnCo_2_O_4_/NF exhibits heterostructures with quadruple hierarchy, including a macroporous NF scaffold, MnCo_2_O_4_ array, ultrathin MnO_2_ nanosheets and ordered mesopores within MnO_2_ nanosheets. With the characteristic hierarchical structure, the MnO_2_/MnCo_2_O_4_/NF electrode demonstrates high current responses in UOR tests (1000 mA cm^–2^ g^–1^ at 1.7 V vs. RHE). Tsiakaras et al. [41] revealed promising catalytic performance can be achieved for Ni/V_2_O_3_/N-doped carbon/NF heterostructured samples (Ni@C–V_2_O_3_/NF, Figure 6a). The 3D hierarchical architecture not only facilitates electron transfer but also promotes gas and electrolyte diffusion (Figure 6b). As a result, UOR performance can be improved. Cao et al. used a two-step hydrothermal reaction method to prepare NiS@Ni_3_S_2_/NiMoO_4_ with NiMoO_4_ nanosheets grown on NiS@ Ni_3_S_2_ nanorod arrays (Figure 6c,d) [42]. Compared to NiS@Ni_3_S_2_ (8.8 mF cm^−2^) and NiMoO_4_ (6.3 mF cm^−2^), double-layer capacitance (C_dl_) reaches 18.3 mF cm^−2^ for NiS@Ni_3_S_2_/NiMoO_4_ (Figure 6e), reflecting more abundant active sites for the heterostructured sample. Moreover, the presence of different domains at the interfaces could regulate the surface charge state of NiMoO_4_, where oxygen acts as a nucleophilic region and molybdenum serves as an electrophilic region. The carbonyl group in urea molecules tends to adsorb on the former, while the amino group favors adsorption on the latter. As a result, the C-N bond breaking can be facilitated and urea molecules can thus be effectively decomposed (Figure 6f). Therefore, the prepared NiS@Ni_3_S_2_/NiMoO_4_ demonstrates the lowest Tafel slope (30 mV dec^−1^) compared to NiS@Ni_3_S_2_ (40 mV dec^−1^) and NiMoO_4_ (53 mV dec^−1^) counterparts, showing improved reaction kinetics in UOR application. Chen et al. used the same concept when preparing heterostructured Ni_3_S_2_/Ni_3_P catalyst [43]. The Ni_3_S_2_ has electron-donating ability, while the Ni_3_P is capable of electron-withdrawal. Thus, two adjacent active sites at the heterojunction interfaces can facilitate the adsorption and decomposition of urea molecules, leading to enhanced UOR performance. 

Lv et al. [44] synthesized 3D heteroporous MoS_2_/Ni_3_S_2_ directly on NF substrate. Taking advantage of the heteroporous structure, the MoS_2_/Ni_3_S_2_ electrode provides abundant active sites and plentiful microchannels for facilitated mass transport. Therefore, the catalytic performance of MoS_2_/Ni_3_S_2_ is greatly improved with high catalytic current of ~600 mA cm^−^^2^ at 1.45 V (vs RHE). Yin et al. [45] prepared FeNi_3_/MoO_2_ heterojunction nanosheet arrays on NF (Figure 6g,h), serving as a bifunctional catalyst for the UOR and HER. In addition to the merit of rich active sites, the strong interaction between FeNi_3_ and MoS_2_ at heterojunction interfaces could cause electron redistribution which promotes the decomposition of urea. As a result, heterostructured FeNi_3_/MoO_2_ catalyst demonstrates promising UOR performance, as evidenced by high catalytic current and turnover frequency (TOF, Figure 6i). Benefitting from rich active sites, regulated electronic structure and promoted mass transport, rational design of heterogeneous catalysts can boost UOR performance, thus showing great potential for overall urea electrolysis.

#### 2.2.2. Enhancing Intrinsic UOR Catalytic Activity

##### Crystallographic Effects of Ni(OH)_2_

**Figure 7:**
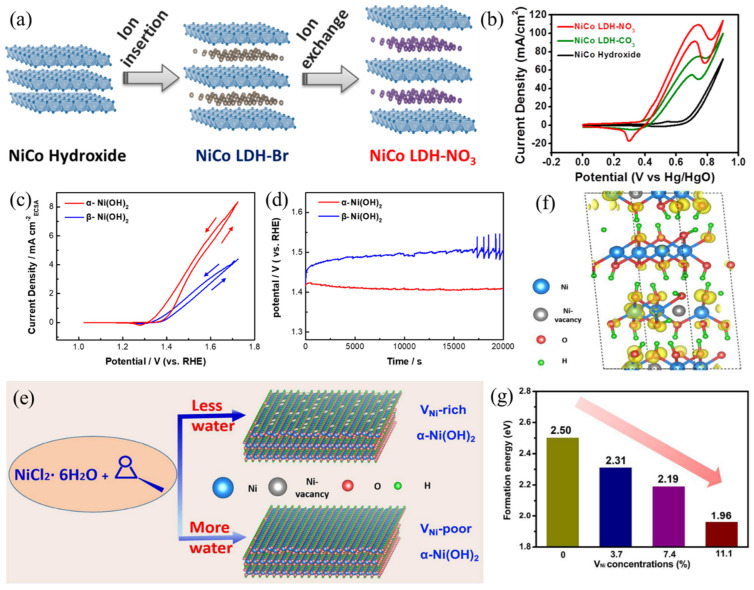
(**a**) Illustration of the synthetic process and (**b**) CV curves of NiCo LDH samples in 1 M KOH with 0.33 M urea; comparison of (**c**) CV curves and (**d**) stability tests of α- and β-Ni(OH)_2_ in 1 M KOH with 0.33 M urea; (**e**) illustration of the preparation of Ni vacancies in α-Ni(OH)_2_; (**f**) DFT simulation at the Fermi level induced by Ni vacancies and (**g**) the calculated formation energies acquired to form active γ−NiOOH; (**a**,**b**,**e**–**g**) Reprinted with permission from Refs. [46,48]. (**c**,**d**) Reprinted with permission from Ref. [47].

Yang et al. [46] revealed that the interlayer distance of NiCo layer double hydroxide (LDH) plays an important role in electro-oxidation of urea. Brucite NiCo LDH was first synthesized by a chemical precipitation method. The as-prepared material was characterized as a layered structure with an interlayer distance of 4.7 Å. Through an ion exchange treatment, CO_3_^2−^ or NO_3_^−^ anions were intercalated into the gallery space of LDH (Figure 7a), leading to expanded interlayer spacing (7.6 Å for NiCo LDH-CO_3_ and 8.6 Å for NiCo LDH-NO_3_). Their results demonstrate that the larger interlayer distance in NiCo LDH renders better catalytic performance. Among them, NiCo LDH-NO_3_ exhibits the lowest onset potential, highest current responses and highest faradaic efficiency (Figure 7b), indicating that expanding the interlayer distance of NiCo layer double hydroxide (LDH) can effectively boost intrinsic UOR catalytic activity. In addition to regulating the interlayer distance of β phase LDH, Wu et al. [47] further revealed the crystallographic effects of Ni(OH)_2_ on catalytic activity toward urea oxidation. Compared to one electron transfer in β−NiOOH/β−Ni(OH)_2_, γ−NiOOH/α−Ni(OH)_2_ redox transition exhibits better electrochemical activity due to more than one electron transfer (~1.5−1.7). By normalizing the current responses with respect to the ECSA values, the intrinsic catalytic performance of α− and β−Ni(OH)_2_ was evaluated and compared (Figure 7c). The results show that the as-prepared α−Ni(OH)_2_ electrode demonstrates improved UOR performance, that is, higher current responses (3.0 vs. 1.6 mA cm_ECSA_^−2^), lower Tafel slope (89 vs. 121 mV dec^−1^) and higher apparent reaction rate constant (6.13 × 10^3^ vs. 1.58 × 10^3^ mol^−1^ s^−1^). Moreover, the α-Ni(OH)_2_ is capable of stable UOR application which preserves its pristine crystal structure, while severe loss of active material can be observed for β−Ni(OH)_2_ (Figure 7d).

Song et al. [48] demonstrated the importance of Ni vacancies in UOR application. Experimentally, propylene oxide (PO) and ethanol were used to control the nucleation and growth of α-Ni(OH)_2_ (Figure 7e). By changing the volumetric ratio between ethanol and water, different amounts of Ni vacancies in α-Ni(OH)_2_ can be obtained. Their results indicate that with richer Ni vacancies existing in α-Ni(OH)_2_, higher UOR catalytic activity is achieved. DFT calculations were further employed to understand this phenomenon. With increased Ni vacancies, α-Ni(OH)_2_ exhibits improved intrinsic conductivity which facilitates the electron transfer process. Moreover, the formation energies required to form active γ-NiOOH can be greatly reduced, promoting the electrochemical oxidation process (Figure 7f,g). Combining the experimental and computational results, creating Ni vacancies using a defect engineering strategy was confirmed to endow α-Ni(OH)_2_ with superior electrocatalytic activity towards the UOR. The above examples reveal that both bulk crystal structures and atomic-level engineering of LDH can enhance UOR performance.

##### Heteroatom Doping

**Figure 8:**
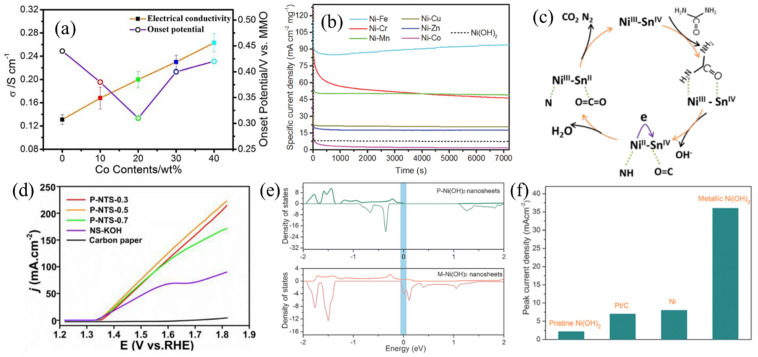
(**a**) Cobalt content dependence of electrical conductivity and onset potential; (**b**) chronoamperometric curves of Ni-M LDH in 1 M KOH and 0.33 M urea; (**c**) proposed reaction mechanism and (**d**) LSV curves of NiSn sulfide catalysts in 1 M KOH with 0.33 M urea; (**e**) density of states of S-doped and pristine Ni(OH)_2_ and (**f**) comparison of peak current densities obtained in 1 M KOH and 0.33 M urea. (**a**) Reprinted with permission from Ref. [54]. (**b**–**d**) Reprinted with permission from Refs. [55,57]. (**e**,**f**) Reprinted with permission from Ref. [58].

Incorporating a proper amount of a second metallic element to Ni-based catalysts has been demonstrated as a promising approach for modulating the electronic structures of electrocatalysts, which can effectively boost intrinsic UOR catalytic activity. To date, binary Ni-Co [49], Ni-Cr [50], Ni-Mn [51], Ni-Fe [52] and Ni-Pd [53] have been successfully prepared, and their UOR performance was confirmed to surpass pure Ni counterparts. Wu et al. [54] prepared a series of Ni−Co bimetallic hydroxide catalysts with different Co contents (0%, 10%, 20%, 30% and 40%). Their UOR performance was examined in 1 M KOH with 0.33 M urea, revealing that 20% Co-doping would be the optimal condition. Electrical conductivity is 0.131 S cm^−1^ for pure Ni catalyst, while Co-doping effectively improves the electrical conductivity of the catalyst. This means incorporating Co can facilitate the electron transfer process in UOR operation, thereby decreasing the onset potential for electro-oxidation of urea (Figure 8a). With higher Co content in the Ni−Co bimetallic hydroxide, higher electrical conductivity is obtained. For the 40% Co-doping sample, its electrical conductivity reached 0.201 S cm^−1^. Nevertheless, Co has much lower catalytic activity compared to Ni. As such, incorporating high Co content would decrease the available Ni active sites for urea oxidation. Thus, an optimal condition for Co content (20%) in Ni−Co bimetallic hydroxide was found, exhibiting reduced overpotential (130 mV less) than the pure Ni catalyst. Moreover, for preparing Ni-Co bimetallic hydroxide with the electrodeposition method, the introduction of Co could alter the surface morphology of the electrodes, demonstrating distinctive UOR performance [49]. 

Schechter et al. [50] found that introducing Cr could effectively boost the reaction kinetics toward urea oxidation with a reduced Tafel slope and charge transfer resistance. The binary NiCr electrocatalyst with 40% Cr shows the highest catalytic activity (2933 mA mg_Ni_^−1^), which is 3.6 times higher than that of the pure Ni controlled sample. Tao et al. [55] systematically compared the UOR performance of Ni−M LDH (M = Cr, Mn, Fe, Co, Cu and Zn) electrocatalysts (Figure 8b). The results indicate that Ni-Fe LDH exhibits the highest activity of ~95 mA cm^−2^ mg^−1^ at 1.54 V (vs. RHE), which is ca. 10 times better than that of pure Ni(OH)_2_. In addition, Liu et al. [56] prepared electrospun ternary Co-Ni-Cr nanoparticles on carbon nanofibers. In their results, ternary catalysts exhibit better catalytic performance compared to single and binary counterparts, showing potential as ternary catalysts for the UOR. Recently, Lu et al. [57] developed a Ni−Sn binary catalyst, which possesses dual-active sites for adsorbing urea molecules. According to the orbital symmetry matching principle, amino groups can form σ bonds with the e_g_ orbital of Ni atoms, whereas carbonyl groups can form p-π bonds with Sn atoms. In this regard, Ni and Sn dual-active sites point at amino and carbonyl groups, respectively. The self-driven electron transfer between Ni and Sn is beneficial for weakening the C−N bond in urea molecules. The proposed reaction mechanism is displayed in Figure 8c. By using this novel material design, Ni-Sn binary catalyst exhibits promising catalytic activity with low onset potential (1.36 V vs. RHE) and high mass activity (180 A g^−1^ at 1.5 V vs. RHE), as shown in Figure 8d. 

Based on the above results, strategic design of binary/ternary catalysts could enable promoted reaction kinetics for electro-oxidation of urea, showing great potential for urea electrolysis. It is worth mentioning that incorporating S and Se has also been reported as a promising strategy for boosting UOR performance. For instance, S-doped Ni(OH)_2_ demonstrates metallic property (Figure 8e), which effectively promotes electron transport resulting in high UOR performance (Figure 8f) [58]. Due to the better conductivity of S and Se (with respect to O), nickel sulfides [59,60,61] and nickel selenides [8,62,63] have been widely employed as electrocatalysts for the UOR. In addition, N-doping has been shown to promote the formation of Ni^3+^ active sites for electro-oxidation of urea [64]. Additionally, the N dopants effectively weaken binding strength between the CO_2_ molecule and the Ni^3+^ active site, alleviating CO_2_ poisoning. Combining these features, N-doping is recognized as a promising approach for obtaining efficient electrocatalysts for the UOR. Table 1 summarizes the UOR performances of various Ni-based catalysts reported in literature.

## 3. Ni-Based Electrocatalysts for HER in Alkaline Medium

The HER is a half-reaction of water splitting that converts water into hydrogen (in an alkaline medium). Despite the fact that the process uses basic reactants and just two electrons for each hydrogen molecule, the many elemental reactions lead to accumulation of energy barriers, resulting in sluggish kinetics. Depending on the reaction circumstances, the HER can be represented in a variety of ways, including acidic, alkaline and neutral solutions. The intrinsic feature of catalysts influences the rate-determining stage of the reaction. The Volmer step in alkaline media is the process by which a water molecule is converted into an adsorbed hydrogen atom and a hydroxide anion. Following that, two adsorbed hydrogen atoms are joined to form a hydrogen molecule (Tafel step) or connected with a water molecule to form a hydrogen molecule and a hydroxide anion (Heyrovsky step) [65].

(9) $$H_{2}O+e^{-}\;\to \;H_{\mathrm{ads}}+\;{OH}^{-}\;\;\;\mathrm{Volmer}$$

(10) $$2H_{\mathrm{ads}}\;\to \;H_{2}\;\;\;\;\;\;\;\;\mathrm{Tafel}$$

(11) $$H_{\mathrm{ads}}+H_{2}O+e^{-}\;\to \;H_{2}\;+\;{OH}^{-}\;\mathrm{Heyrovsky}$$

In contrast to the acidic state, the Volmer step in the alkaline condition includes the adsorption of water and the desorption of the hydroxide anion, demonstrating that different reaction processes can have a significant impact on the kinetic characteristics of electrocatalysts. As an introduction to nickel-based catalysts, we first discuss the most recent developments in nickel-based HER catalysts, covering numerous nickel-based compound categories, before providing some insight into the future of nickel-based HER catalysts by examining the association between catalytic activity and chemical composition or catalyst active site.

### 3.1. Metallic Ni-Based

**Figure 9:**
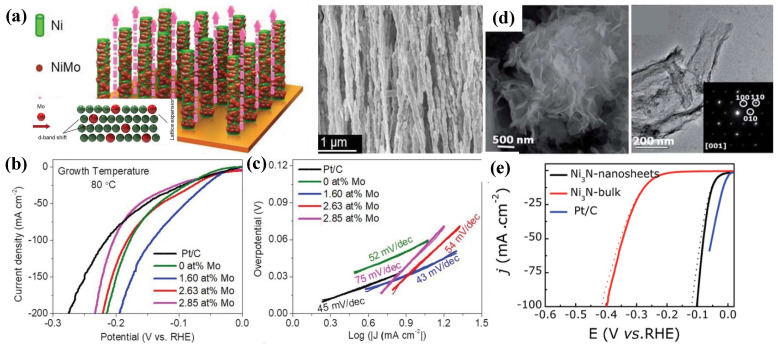
(**a**) Illustration of a schematic fabrication of NiMo nanowire arrays via magnetic field-assisted growth and SEM image. (**b**) LSV curves and (**c**) corresponding Tafel plot of NiMo nanowire arrays, NiMo-65; reprinted with permission from Ref. [66]. (**d**) SEM and TEM images and (**e**) polarization curves of Ni_3_N nanosheets with a scan rate of 2 mV s^−1^; reprinted with permission from Ref. [67].

Because of its high catalytic activity, cost-effectiveness and excellent stability, metallic Ni is used as an electrocatalyst for the HER in the study of electrocatalytic water splitting. Notably, the Ni nanostructure demonstrated outstanding HER activity. However, the unsatisfactory stability of Ni catalysts caused by nickel hydride generation in the HER process impedes the reaction’s rapid progress. Alloying is an effective method for modifying the properties of the catalyst surface. Nairan et al. [66] had demonstrated the excellent HER activity of NiMo alloy nanowire arrays (Figure 9a), prepared through a magnetic field assistance in an aqueous-based method. This NiMo alloy demonstrates an extremely low overpotential of 17 and 98 mV at 10 and 400 mA cm^−2^ (Figure 9b), respectively, in alkaline condition, which outperforms commercial Pt/C. The study shows that the lattice distortions are caused by Mo incorporation and increased interfacial activity. The synergistic effect of Ni and Mo led to the optimization of H_adsorption_ energy and a large number of MoNi_4_ active sites on the surface of nanowires; both contributed to substantially increased catalytic activity [66]. On the other hand, metallic Ni has been shown to enhance the HER activity of transition-metal nitrides that are involved in facilitating electron transfer during HER catalysis. A study by Gao et al. [67] found that developing an atomically thin metallic Ni_3_N 2D nanosheet (Figure 9d) exhibits remarkable HER performance, affording an ultralow overpotential of 100 mV at a current density of 100 mA cm^−2^ (Figure 9e). Later, the theoretical calculations showed that carrier concentration and electrical conductivity of 2D metallic Ni_3_N nanosheets were successfully improved. Because of the Ni-N co-effect, Ni atoms on the N-Ni surface with surrounding N atoms have the smallest Δ*G*_H*_ of 0.065 eV, acting as the most active HER sites in Ni_3_N. 

### 3.2. Ni-Based Oxide/Hydroxide

**Figure 10:**
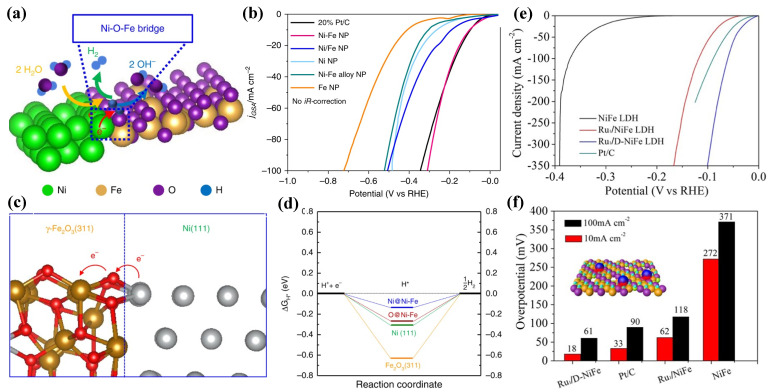
(**a**) Schematic representation. (**b**) HER-LSV curves of the Ni-Fe Janus nanoparticles, theoretical comprehension. (**c**) Ni-Fe heterojunction interface structure that has been optimized. (**d**) Standard free energy diagram of the HER process on the surfaces of Fe_2_O_3_(311) and Ni(111) in the Ni-Fe heterojunction. (**e**) HER polarization curves, reprinted with permission from Ref. [69], and (**f**) overpotentials at typical current densities of various LDHs, reprinted with permission from Ref. [70].

Among electrocatalysts, Ni-based oxide/hydroxide exhibits excellent electrocatalytic properties for overall urea splitting. Recent research has shown that the present M-OOH metal ions with low 3D energy states are the true UOR active sites, boosting metal–oxygen interaction and facilitating adsorption/desorption of intermediate products [68]. Thus, oxide/hydroxide electrocatalysts for urea electrolysis are the most favorable. Recently, as reported by Suryanto et al. [69], a Janus Ni-Fe nanoparticle (Ni-Fe NP, Figure 10a) with a Ni metal domain linked to γ-Fe_2_O_3_ that forms a heterojunction/interface displayed exceptional HER catalytic activity compared to the standard Pt/C catalyst. To achieve a current density of 10 mA/cm^2^, Ni-Fe NPs only require a very low HER overpotential of 100 mV (without iR-corrections) in 1 M KOH solution (Figure 10b). Later on, the DFT simulations (Figure 10c,d) suggested that the Ni-O-Fe bridge at the Ni-γ-Fe_2_O_3_ interface changes the Gibbs free energy of the adsorption of the intermediate H atoms (ΔG_H__*_), thus enhancing the performance of HER catalysis. Surprisingly, overpotential for the OER is also reduced as a result of the multi-site features developed at the interface. This study shows that introducing asymmetry into an electrocatalyst structure results in an unparalleled synergistic impact for electrocatalysis, which overcomes the practical constraint of Ni-Fe-mixed oxides for total water electrolysis (their low HER activity) by using this technique. They also compared the redox behavior of Ni-Fe NPs with a physical mixture of Ni NPs and Fe NPs (denoted as Ni/Fe NPs) and a Ni-Fe alloy mixture to investigate this role. Their findings show that overpotentials of 112 and 307 mV are required for Ni/Fe NPs and Ni-Fe alloy NPs, respectively, to achieve 10 mA cm^−2^ (Figure 10b), which are significantly higher than those required for Ni-Fe NPs, validating the role of the Ni-γ-Fe_2_O_3_ interface in HER performance. 

While NiFe-layered double hydroxide (NiFe-LDH) offers great potential as a bifunctional electrocatalyst for simultaneously catalyzing the HER and OER in alkaline solutions, the hydrogen binding property on the Fe^3+^ center is rather sluggish, resulting in a high kinetic energy barrier for the Volmer step and sluggish HER kinetics under alkaline conditions. To break through this barrier, atomic-level control of active sites is required. As a result, rational design of single atom catalysts on common LDH is crucial. Zhai et al. [70] demonstrated that a single-atomic-site ruthenium catalyst sustained on defective NiFe-LDH had excellent HER and OER performance. Although Ru and NiFe-LDH are considered active OER catalysts, as-synthesized Ru_1_/D-NiFe LDH (Figure 10e) achieves a current density of 10 mA cm^−2^ at an ultralow overpotential of 18 mV and a high turnover frequency of 7.66 s^−1^ at an overpotential of 100 mV (45 times higher than that of commercial Pt/C catalyst) for HER 1 M KOH electrolyte. DFT simulations show that Ru1/D-NiFe LDH optimizes the favorable control of H adsorption energies for the HER and increases O-O coupling due to the presence of Ru-O moieties.

### 3.3. Ni-Based Dichalcogenides and Compounds 

**Figure 11:**
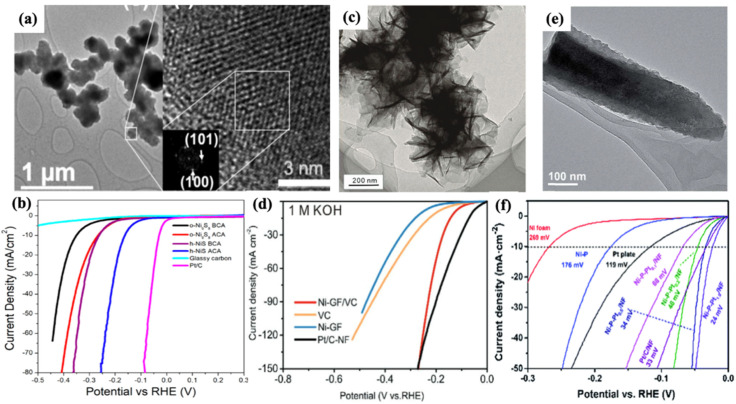
(**a**) SEM and HRTEM image of h-NiS. (**b**) HER-LSV curves of *o*-Ni_9_S_8_ and *h*-NiS, reprinted with permission from Ref. [73]. (**c**) HRTEM images and (**d**) HER performance of the Ni-GF/VC catalyst, reprinted with permission from Ref. [71]. (**e**) TEM image and (**f**) LSV curves of Ni-P-Pt/NF catalysts for the HER in 1 M KOH aqueous solution with iR correction, reprinted with permission from Ref. [76].

Because of their high OER performance in alkaline media, non-noble-metal compounds, such as carbides, phosphides and chalcogenides, have drawn enormous attention recently. Among those, transition metal-based carbides and phosphides have been known to possess “Pt-like behaviour” for the HER in alkaline media, including the most intriguing candidates—nickel-based compounds [71,72]. However, only limited research has concentrated on HER electrocatalysis in alkaline media, with the majority of the existing research concentrating on acidic media. Crystalline nickel sulfides are particularly promising catalysts, as reported by Silva et al. [73] who later found that their performance correlates to their crystalline structure. Both of the as-synthesized phases of nickel sulfide, orthorhombic (o-Ni_9_S_8_) and hexagonal nickel sulfide (h-NiS), showed excellent HER activity in an alkaline medium, even surpassing W_2_C and Mo_2_C (Figure 11b). The sulfur precursor selected is crucial in controlling the crystal structure, size and morphology of the resulting materials. The nanometric features on the surface of h-NiS nanoparticles raise surface roughness, thus leading to better HER activity than orthorhombic nickel sulfide (o-Ni_9_S_8_). Similarly, the transition metal carbides (TMC) are endowed with the characteristic resembling Pt, due to the hybridization of metal d-orbitals with carbon s- and p-orbitals that results in a broadened metal d-orbital in TMCs. Their performance is still hampered, however, due to their strong interaction with hydrogen. To overcome these challenges, Yang et al. [71] proposed Ni activation of TMCs via adsorbed nickel atoms on the TMC surface (Ni/TMC), as shown in Figure 11c. Following the addition of nickel adsorbed atoms, binder-less Ni-GF/TMCs (Ni foam coated with graphene–vanadium carbide) exhibit superior HER performance in both alkaline and acidic media, as well as excellent stability towards the HER. The exceptional performance is due to the unique structural and electrical properties of Ni-activated TMCs. The introduction of absorbed nickel atoms on the surface of TMCs effectively optimizes the d-electron structure, leading to an enhanced number of active sites and the enhanced intrinsic catalytic activity of TMCs. Thus, a sharp decrease in both overpotentials and Tafel slopes of the Ni/TMC catalysts for the HER was observed, with values of 128 mV at 10 mA cm^−2^ in 1 M KOH and 111 mV in 0.5 M H_2_SO_4_, respectively (Figure 11d). 

In particular, nickel phosphides (Ni-P) have been investigated as advanced HER electrocatalysts in recent years due to their unique electronic structure, low cost and good corrosion resistance [74]. It was recently demonstrated that combining Pt with other metals can improve its performance by enhancing water dissociation and catalytic activity [75]. As a result, Xia et al. [76] identify that P–Pt and Ni–Pt interactions will tune Pt’s electronic and catalytic properties for the HER. By synthesizing Ni-P-Pt/NF catalyst (nickel phosphide contains a trace amount of Pt by chemical adsorption) as shown in Figure 11e, it exhibits very low overpotential, high stability and fast reaction kinetics, with an overpotential of only 34 mV at a current density of 10 mA cm^−2^ (Figure 11f) and a Tafel slope of 31 mV dec^−1^. Theoretical calculations indicate that replacing Ni atoms with Pt moves the Ni-P–Pt in the volcano plot closer to Pt due to improved hydrogen adsorption and catalytic activity. In addition, the excellent performance can be attributed to the synergistic effect between the Ni-P and Pt, which results in a stronger reduction power of Pt, better reaction kinetics and hydrogen adsorption. The presence of iron in a bimetallic catalyst system, according to the authors, can introduce extra structural vacancies which increase the activity of FeNi-MOFs.

### 3.4. Ni-Based MOFs

**Figure 12:**
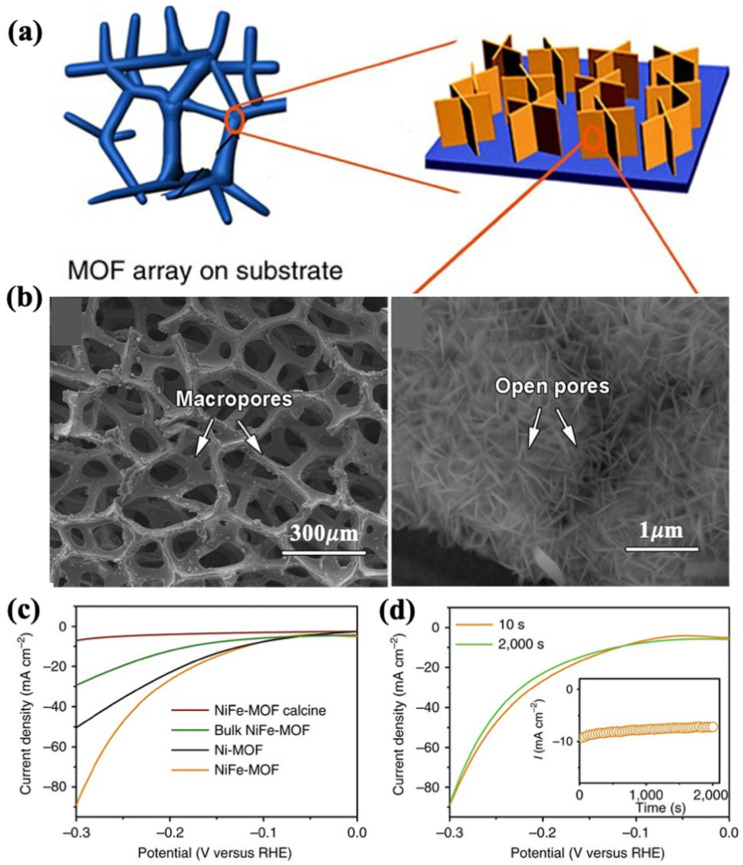
(**a**) Schematic illustration. (**b**) SEM images of the NiFe-MOF array. (**c**) LSV plots obtained with a NiFe-MOF, bulk NiFe-MOF, Ni-MOF and calcined NiFe-MOF for the HER at 10 mV^−1^ in 0.1 M KOH. (**d**) LSV of NiFe-MOF for HER before and after chronoamperometric testing for 2000 s at 0.2 V (versus RHE) in 0.1 M KOH; the inset of (**d**) shows corresponding chronoamperometric profile. Reprinted with permission from Ref. [79].

Metal organic frameworks (MOFs) have recently been regarded as a prominent class for developing uniformly distributed metal nanostructures over ordered carbon matrices as an efficient electrocatalyst for the HER [77,78]. Due to their powerfully built and adaptable network, pristine MOFs or MOFs used as supporting frameworks can be used to halt, scatter and remove external species that are catalytically active. MOFs, in whatever form, can be used as a precursor to develop a wide range of metal components or metal/carbon composites with structured elemental composition and structure. This would help boost the implementation of either the guest material or the host. A study regarding pristine MOFs by Duan et al. [79] reported on in situ growth of ultrathin nanosheet arrays of bimetallic FeNi-MOFs that demonstrated superior electrocatalytic activity for the HER in a basic medium (0.1 M KOH) as illustrated in Figure 12a. This FeNi-MOFs is porous-rich nanosheet, with a pore ranging from 200–400 μm (Figure 12b). At a current density of 10 mA cm^−2^, FeNi-MOF exhibited overpotential of 134 mV (Figure 12c) and demonstrated stable activity at 200 mV for up to 2000 s (Figure 12d). The presence of iron in a bimetallic catalyst system, according to the authors, can introduce extra structural vacancies which increase the activity of FeNi-MOFs. Furthermore, because the catalyst grows directly on the nickel foam, there is no need for additional binders in electrode preparation. Besides that, the porous structure of the Ni foam as a substrate improves catalyst performance by altering electrolyte and product mass transport. 

### 3.5. Single-Atom Ni

**Figure 13:**
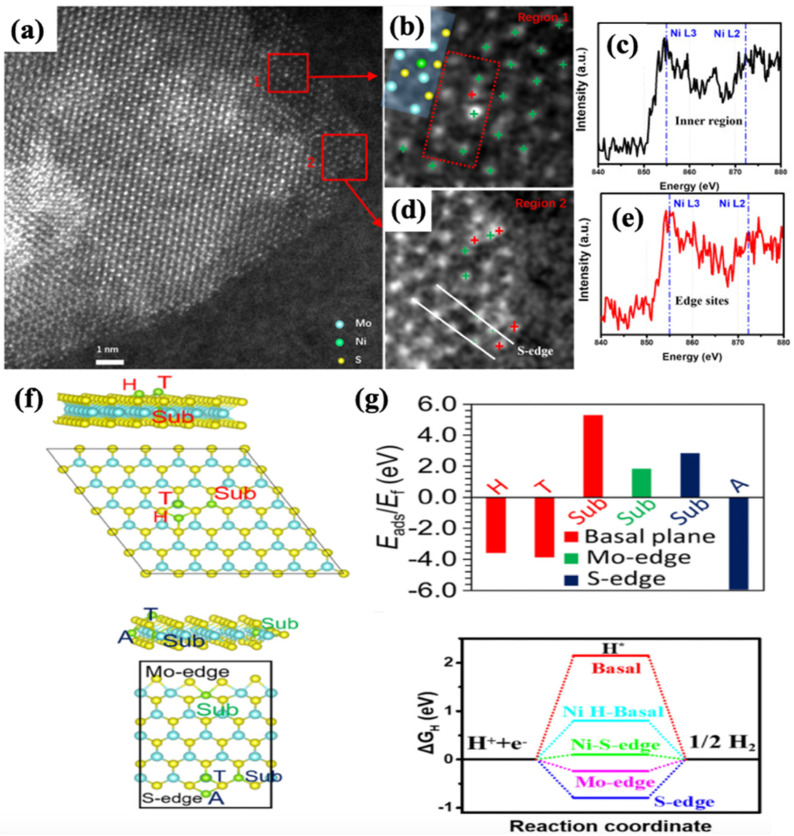
(**a**) STEM images of NiSA-MoS_2_/CC and the magnified images of (**b**) region 1 and (**d**) region 2; Ni atoms are represented by red crosses and Mo atoms are represented by green crosses. EELS spectra of Ni for (**c**) inner region 1 and (**e**) edge sites at region 2. (**f**) Atomic-level configuration of the absorption of Ni on the basal plane for both the top of the Mo sites (T) and the center of the hexagon sites (H); Mo atom substitution in the MoS_2_ monolayer with Ni and the configuration of Ni absorption on the S-edge at T and A sites; and Mo atom substitution at the Mo-edge and S-edge with Ni. (**g**) Ni adsorption energy on MoS_2_ and Mo atom substitution energy in MoS_2_, as well as H* adsorbed Gibbs free energies at the basal plane, the Mo edge and the S edge either with or without Ni adsorption/substitution. Reproduced from Elsevier [81].

Reducing the size of nanoparticles, in particular to the atomic scale, can theoretically allow for maximum atom utilization efficiency as well as high activity and selectivity in the catalytic reaction. Electronic perturbations, such as shifts in the energy of the d-band center, can affect the chemical and catalytic properties of single atoms and supports due to their electronic interaction. Thus, the energy level of the d-band center is correlated to the binding of hydrogen adsorption to the surface of the catalyst, allowing for improved hydrogen adsorption by regulating the energy level of the d-band center, as occurs with heteroatom doping [80]. As a result, single-atom catalysts with high atom utilization and unique electronic structures have received a lot of attention. Several Ni single-atom catalysts with high catalytic efficiency have been reported since then for the HER. Wang et al. [81] reported a single Ni atom decorated on MoS_2_ (Figure 13a) that exhibited exceptional HER performance in both alkaline and acidic medium. As single Ni atoms were incorporated into the MoS_2_ S-edge and H sites of the basal plane, HER activity was significantly increased when compared to pure MoS_2_/CC and Ni cluster-decorated MoS_2_/CC. Some embedded foreign atoms in the atomic column of MoS_2_ are identified by evaluating the atomic structure (Figure 13b,c). Area 1 (Figure 13b), the magnified picture of the Ni atom from Figure 13a, revealed that the single Ni atom is located atop a hexagonal site of the basal plane, specifically the H-basal site. 

Scanning transmission electron microscopy (STEM) demonstrates that the Ni single atoms were attached to the basal plane’s S-edge and H sites. The corresponding electron energy loss spectroscopy (EELS) spectra of these sites identify the appearance of Ni peaks at 855 eV, indicating that the embedded atoms are Ni atoms (Figure 13d). According to DFT calculations, the decorated Ni atoms on the S-edge and H site of the basal plane can control the adsorption behavior of H atoms and, hence, HER activity (Figure 13f,g). 

**Figure 14:**
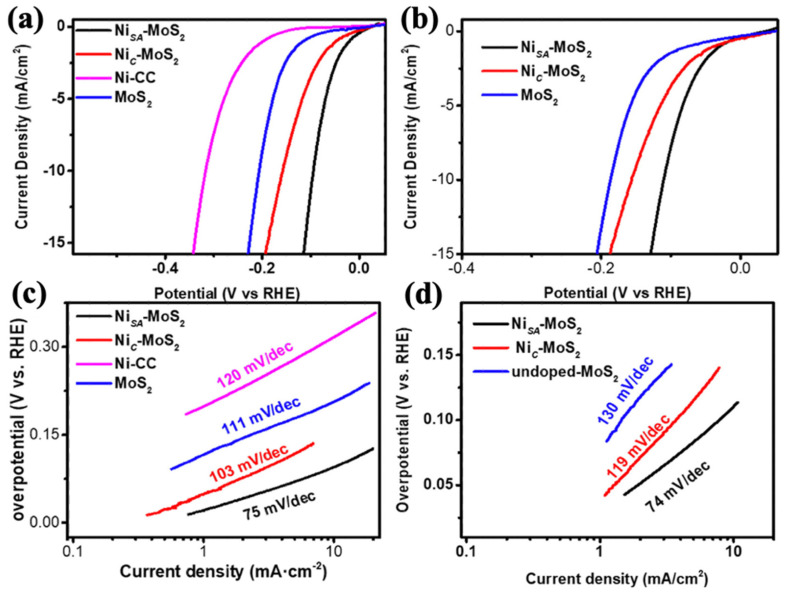
Polarization curves of NiSA-MoS_2_/CC catalyst in (**a**) 1 M KOH and (**b**) 0.5 M H_2_SO_4_ solution with a scan rate of 5 mV s^−1^ and (**c**,**d**) the corresponding Tafel plots. Reproduced from Elsevier [81].

As a consequence, low overpotentials of 98 mV and 110 mV in 1 M KOH and 0.5 M H_2_SO_4_, respectively, at current density of 10 mA cm^−2^ (Figure 14a–d) are afforded. In summary, single Ni atom decorating has no effect on the catalytic behavior of the Mo-edge, but it does boost the catalytic activity of S-edges considerably. 

### 3.6. Ni-Based Heterostructure

**Figure 15:**
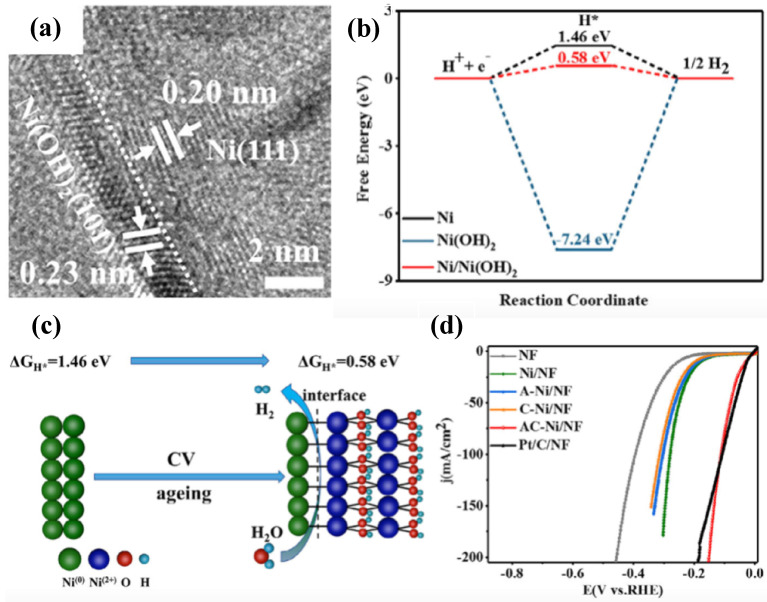
(**a**) HRTEM image of the AC-Ni/NF sample and (**b**) calculated free energy for atomic hydrogen adsorption on Ni, Ni(OH)_2_ and Ni/Ni(OH)_2_. (**c**) Mechanism for the enhanced HER on the Ni/Ni(OH)_2_ heterostructure. (**d**) Polarization curves of AC-Ni/NF and contrast samples at a scan rate of 2 mV s^−1^ in 1M KOH. Reprinted with permission from Ref. [82].

While the HER comprises numerous adsorption/desorption processes involving many species in alkaline conditions, catalysts with single active elements may be restricted in their ability to meet the necessary adsorption/desorption energies of each species, including OH^−^ and H_2_. Incorporating a second component in an extensive junction with the single-component Ni catalyst to generate a heterostructured interface may provide additional chances for tailoring adsorption/desorption energies, resulting in more active catalysts. Furthermore, various material components can be concurrently included to promote the adsorption/desorption of these various particles. A number of similar publications have recently been reported. Lai et al. [82] reported on a Ni/Ni(OH)_2_ heterostructure on a Ni foam substrate using simple electrodeposition and aging (Figure 15a). The Cyclic Voltammetry assisted (CV-treated) electrodeposited Ni nanoclusters on nickel foam substrate were denoted as C–Ni/NF and AC–Ni/NF (partially transferred to Ni(0)/Ni(OH)_2_ heterostructure). DFT simulations were used to better investigate the mechanism behind HER activity. The value of ΔG_H*_ decreased from 1.46 eV on Ni(OH)_2_ to 0.58 eV on the Ni/Ni(OH)_2_ heterostructure catalyst, indicating that the H adsorption energy was highest at the heterojunction interface (Figure 15b,c). Thus, in alkaline conditions, this electrocatalyst requires just 30 mV to obtain a current density of 10 mA cm^−2^ (Figure 15d). 

**Figure 16:**
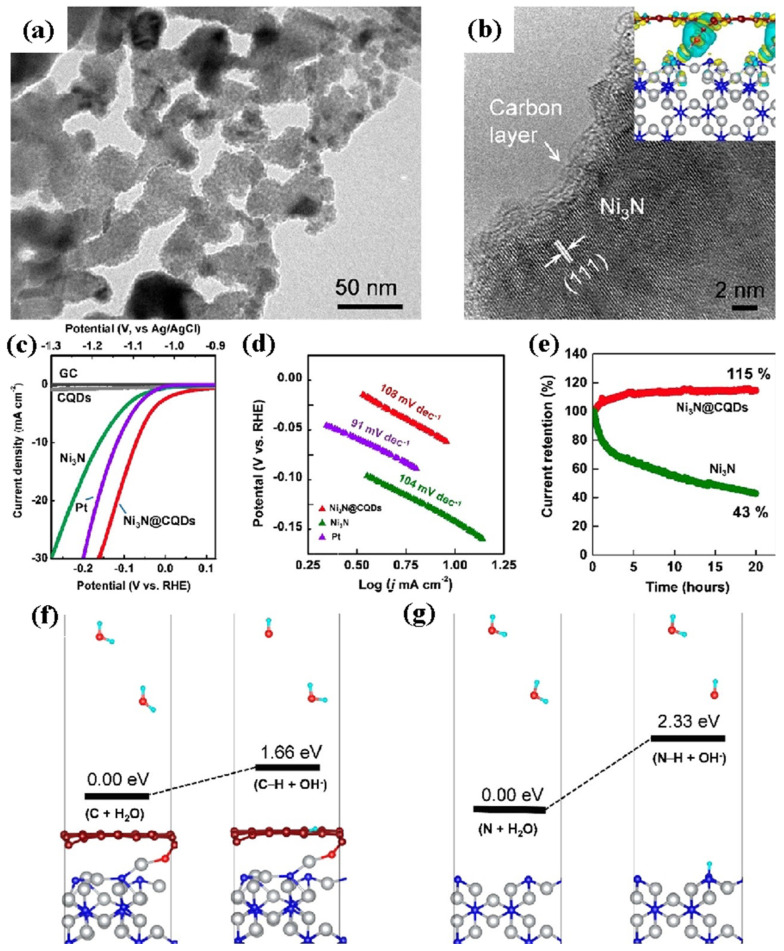
(**a**) TEM image and (**b**) HRTEM image of Ni_3_N@CQDs. (**c**) LSV polarization curves of Ni_3_N@CQDs in comparison with a platinum (Pt) electrode, pristine Ni_3_N, CQDs and a glassy carbon (GC) electrode in a 1 M KOH aqueous solution. (**d**) Tafel slopes. (**e**) Normalized HER amperometric I-t curves of Ni_3_N@CQDs and Ni_3_N at a constant overpotential of 77 mV (−1.1 V vs. Ag/AgCl). (**f**,**g**) Comparison of the HER Volmer reaction step and the resultant binding energies on (**f**) carbon-coated Ni_3_N(110) and (**g**) pristine Ni_3_N(110) surfaces. Reprinted with permission from Ref. [83].

Specific surface modification options, such as carbon introduction, have recently been developed to satisfy the Volmer phase in the alkaline HER process. Zhou et al. [83] attempted to avoid the water dissociation stage by creating a polarized carbon surface on Ni_3_N nanoparticles (Ni_3_N@CQDs) (Figure 16a,b). The carbon-reinforced Ni_3_N was generated by dipping Ni(OH)_2_ in a carbon quantum dot solution and then heating the recovered solids to convert the Ni(OH)_2_ to Ni_3_N. The Volmer step’s activation energy was reduced dramatically on the charge-polarized carbon surface, leading to improved catalytic activity and an overpotential of 69 mV at a current density of 10 mA cm^−2^ in 1 M KOH (Figure 16c) being achieved, which is substantially lower than that of the Pt electrode. Over hours of continuous usage, the carbon covering was found to preserve the inside of the Ni_3_N layer against oxidation/hydroxylation. Table 2 showed the comparison of HER performance for various Ni-based electrocatalysts.

## 4. Bifunctional Electrocatalysts for the UOR and HER

Commonly, bifunctional catalysts comprise two different catalytic sites that are capable of catalyzing two different types of reactions. In urea electrolysis, the bifunctionality of electrocatalysts refers to the ability of catalysts when conducting the redox reaction, such as cathodic HER and anodic UOR, simultaneously [3]. Presently, high-cost, noble, metal-based materials are commercially available electrocatalysts for urea electrolysis and a lot of research studies have therefore been conducted to find low-cost, highly efficient and noble-metal-free bifunctional electrocatalysts [10,84,85]. For several decades, transition metal-based materials have been extensively researched as bifunctional electrocatalysts for water electrolysis, metal-air batteries and urea electrolysis due to their advantageous high electroactivity, high electric conductivity, crystal structures compatible with compositions and morphology and earth abundance [86,87]. In addition, bifunctional electrocatalysts are reducing costs and simplifying the electrode preparation process. The excellent electroactivity of nickel-based materials as bifunctional electrocatalysts in a single electrolytic solution towards urea electrolysis has already been achieved [3]. Herein, we focus on bifunctional nickel-based electrocatalysts for achieving highly efficient urea electrolysis.

### 4.1. Ni-Based Oxides/Hydroxides

**Figure 17:**
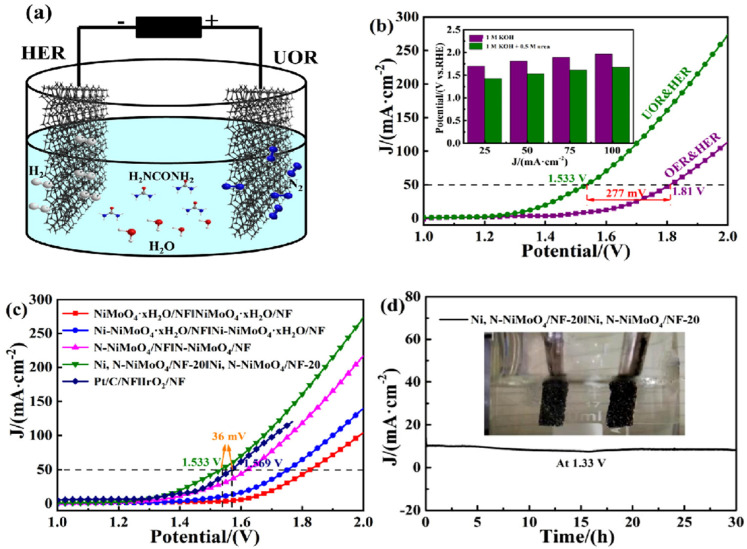
(**a**) Simulation diagram of a two-electrode electrolytic cell. (**b**) Full electrolytic polarization curves of Ni, N-NiMoO_4_/NF-20 in different electrolytes. (**c**) Full electrolytic polarization curves of various two-electrode electrolyzers and (**d**) amperometric I-t curve (inset is the actual two-electrode electrolyzer), reprinted with permission from Ref. [89].

**Figure 18:**
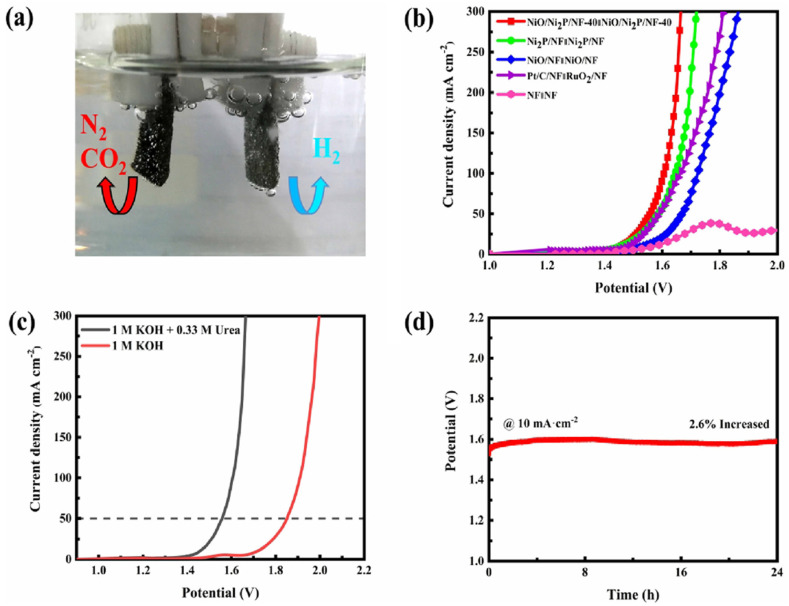
(**a**) Photograph of the urea electrolyzer, (**b**) overall urea electrolytic LSVs in urea electrolyzers containing different electrode pairs, (**c**) LSVs of overall water electrolysis and urea electrolysis and (**d**) chronopotentiometry of NiO/Ni_2_P/NF-40||NiO/Ni_2_P/NF-40 at 10 mA cm^−2^ for urea electrolysis. Reprinted with permission from Ref. [90].

**Figure 19:**
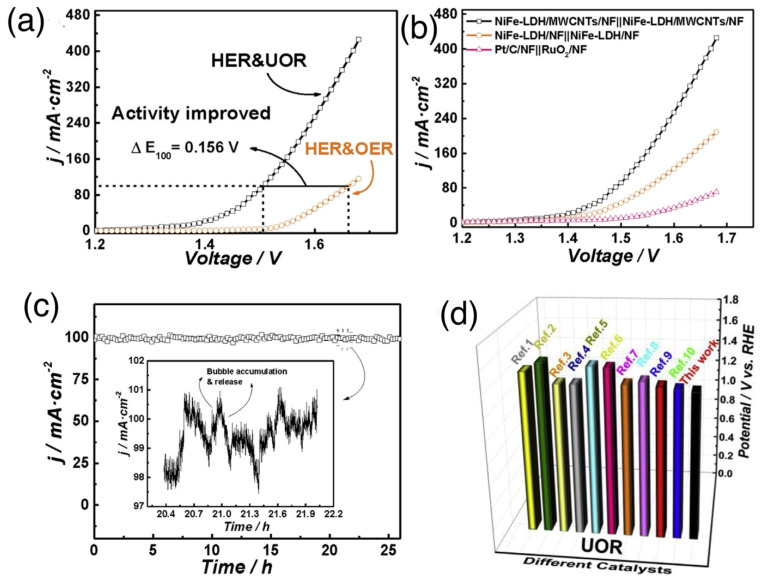
(**a**) LSVs of water electrolysis and water-urea electrolysis, (**b**) LSVs, (**c**) chronoamperometric responses of NiFe-LDH/MWCNTs/NF||NiFeLDH/MWCNTs/NF and (**d**) comparison of the potentials of different catalysts during the UOR. Reprinted with permission from Ref. [91].

**Figure 20:**
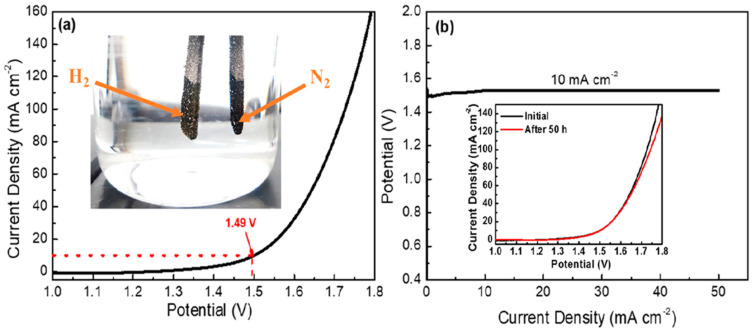
(**a**) Overall electrolysis in a two-electrode system in 1 M KOH with 0.33 M urea and (**b**) long-term stability over 50 h. The inset shows the polarization curves before and after the long-term stability test. Reprinted with permission from Ref. [92].

According to previous reports, high valance Ni^3+^ (in the form of NiOOH) possessing a low 3D energy state is a real active site for the UOR since it can ameliorate metal–oxygen interaction and accelerate the adsorption/desorption of intermediates. Knowing this, Ni-based oxides/hydroxides are mostly presented in the form of NiOOH in alkaline media and thus Ni-based oxides/hydroxides could be more beneficial for efficient bifunctional electrocatalysts towards urea electrolysis [68]. Yu et al. [88] demonstrated a Ni-Mo-O nanorod-derived composite as an efficient bifunctional electrocatalyst for urea electrolyzers. They adopted a gas-selected annealing process and synthesized two different compounds using two various gases of Ar and H_2_, with NF/NiMoO-Ar working as a UOR catalyst and NF/NiMoO-H_2_ working as an HER catalyst. Wang et al. [89] recently reported on electrodeposited Ni and N-doped NiMoO_4_ grown on nickel foam (NF) and investigated their bifunctional electrocatalytic activity for the HER and UOR. Furthermore, they found that the different electrodeposition time of metallic nickel (Ni, N-NiMoO_4_ /NF-x (x = 10, 20, 30 min)) affected their catalytic performance. Figure 17a shows the simulation diagram of the two-electrode alkaline electrolyzer, and its corresponding polarization curves in different electrolytes (Figure 17b) show that the driving voltage needed for urea electrolysis is 1.533 V, which is 277 mV lower than water electrolysis (Figure 17c). The polarization curves of electrolytic cells fabricated using Ni, N-NiMoO_4_/NF-20 catalyst and the cells assembled with other catalysts are exhibited in Figure 17c, with the Ni, N-NiMoO_4_ /NF-20 catalyst demonstrating the best bifunctional electrocatalytic activity among all catalysts, i.e.,176 mV towards the HER and 1.444 V towards the UOR at 100 mA cm^−2^. Figure 17d shows the excellent stability of Ni, N-NiMoO_4_/NF20//Ni, N-NiMoO_4_/NF-20 electrolyzer over a 30-h period. Recently, Xu et al. [90] detailed the use of heterostructured nickel oxide/nickel phosphide nanosheets as a bifunctional electrocatalyst, prepared by an in situ acid etching and gas phase phosphating method. Figure 18a–d displays a photograph of the fabricated urea electrolyzer using a NiO/Ni_2_P/NF-40 electrode and its electrochemical performance in alkaline medium. They developed an electrolysis cell of NiO/Ni_2_P/NF-40||NiO/Ni_2_P/NF-40 by utilizing the same electrocatalyst as an anode and cathode, reaching a current density of 10 mA cm^2^ at 1.457 V, which was lower than the 1.490 V required for the Pt/C/NF||RuO_2_/NF cell. Ni-based layered double hydroxides (LDH) can provide more active sites but their poor conductivity limits their catalytic activity, therefore Wen et al. [91] fabricated nanohybrids of NiFe-LDH/MWCNTs/NF using a one-step hydrothermal process and studied their bifunctional electrocatalyst nature towards water and urea electrolysis. Figure 19a displays the LSV curve of NiFeLDH/MWCNTs/NF||NiFe-LDH/MWCNTs/NF, which reveals water-urea electrolysis (HER and UOR) as more efficient than water electrolysis (HER and OER) by reaching 100 mA cm^2^ (ΔE_100_) at 0.156 V. Only 1.375 V for the UOR and 0.208 V for the HER are required at 50 mA cm^−2^, and the fabricated two-electrode electrolyzers (such as NiFe-LDH/MWCNTs/NF||NiFe-LDH/MWCNTs/NF, NiFe-LDH/NF||NiFe-LDH/NF and Pt/C/NF||RuO_2_/NF which required voltages of 1.344 V, 1.397 V and 1.496 V at 10 mA cm^−2^) demonstrated that NiFe-LDH/MWCNTs/NF||NiFe-LDH/MWCNTs/NF electrolyzer was more efficient than other electrolyzers (Figure 19b). Figure 19d illustrates the required potentials of different catalysts for the UOR compared to their work. Amorphous materials are generally showing more enhanced electrocatalytic activity than crystalline counterparts. Thus, Babar et al. [92] prepared amorphous and porous 2D NiFeCo LDH/NF using the electrodeposition technique and exhibited their bifunctional characteristics toward water and urea electrolysis while demonstrating its electrochemical performance for the UOR and HER in alkaline medium. They showed the excellent performance of urea electrocatalytic cells using NiFeCo LDH/NF as both the cathode and the anode, delivering low cell potential of 1.49 V at 10 mA cm^−2^ and achieving high current density of 100 mA cm^−2^ at 1.72 V (Figure 20a). The long-term stability of NiFeCo LDH/NF catalysts was examined over 50 h in a 1 M KOH with 0.33 M urea solution. Over 50 h of continuous operation, NiFeCo LDH/NF showed a slight change in potential (Figure 20b).

### 4.2. Ni-Based Chalcogenides

**Figure 21:**
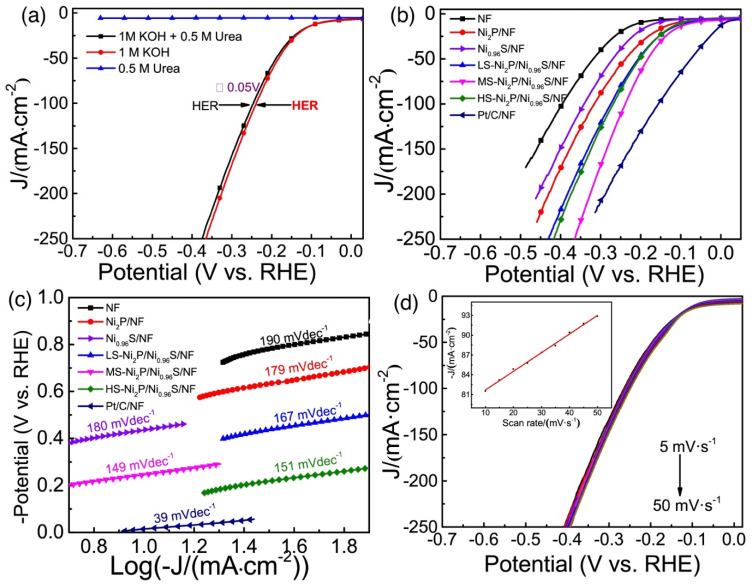
(**a**) LSV curves of MS-Ni_2_P/Ni_0.96_S/NF, (**b**) LSV curves in 0.1 M KOH with 0.5 M urea, (**c**) Tafel plots and (**d**) LSV curves of MS-Ni_2_P/Ni_0.96_S/NF at different scan rates. Reprinted with permission from Refs. [97,98].

Generally, Ni-based chalcogenides (sulfides, selenides and phosphides) have become attractive for urea electrolysis owing to their high catalytic activity, high conductivity and low cost [93]. Bifunctional electrocatalytic activity of Ni-based chalcogenides is designed using strategies such as nanostructure control, composition optimization and heterostructuring [94,95]. Typically, nickel sulfides are of great interest due to their high theoretical catalytic activity and their sensibility to the phase structure. Hydrothermally prepared Ni_3_S_2_/NF nanowire as a bifunctional electrocatalyst for urea electrolysis was reported by Liu et al. [96] and delivers excellent UOR performance (current density of 100 mA cm^−2^ at 0.36 V (vs. SCE) in the electrolyte of 1.0 M NaOH and 0.33 M urea) and HER activity (overpotential of 127 mV dec^−1^ vs. at 10 mA cm^−2^). Finally, the two-electrode system assembled with Ni_3_S_2_@NF can operate at 20 mA cm^−2^ at a cell voltage of only 1.49 V with excellent longevity [96,97]. The use of various heterostructured Ni_2_P/Ni_0.96_S particles with different S/P ratios as a bifunctional catalyst was reported by He et al. [97] and the morphologies varied with S content. LSVs of MS-Ni2P/Ni0.96S/NF in different electrolytes are shown in Figure 21a, indicating that the catalyst had no activity when only urea was present. Figure 21b shows the HER activities of different catalysts in alkaline electrolyte with 1.0 M KOH and 0.5 M urea. The catalytic reaction kinetics were evaluated using the Tafel plots (Figure 21c) for NF, Ni_2_P/NF, Ni_0.96_S, LS-, MS-, HS-Ni_2_P/Ni_0.96_S/NF and referential Pt/C/NF electrodes, and the Tafel slopes were determined as 190, 179, 180, 167, 149, 151 and 39 mV·dec^−1^, respectively. Moreover, a good proportional linear relationship between current density and scan rates at −0.24 V from LSV curves (Figure 21d) has been observed for MS-Ni_2_P/Ni_0.96_S/NF electrode, suggesting high efficiency of charge and mass transfer towards the HER. A two-electrode system (Ni_2_P/Ni_0.96_S/NF||Ni_2_P/Ni_0.96_S/NF) for overall urea-water electrolysis was prepared and required a cell voltage of only 1.453 V to drive current density of 100 mA cm^−2^ in an alkaline medium for both the HER and UOR, which is 186 mV lower than that of overall water splitting. In addition, for achieving a current density of 100 mAcm^−2^, the MS-Ni_2_P/Ni_0.96_S/NF||MS-Ni_2_P/Ni_0.96_S/NF system only requested a cell voltage of 1.453 V, which was lower than other electrolysis systems and is even 240 mV lower than that of the Pt/C/NF||IrO_2_/NF system. The excellent long-term stability of the catalyst was verified with 20 h of urea electrolysis and the current density of the cell was maintained at around 50 mA cm^−2^ and remained close to 90% [97]. Wang et al. [98] synthesized hierarchical coral-like Ni-Mo sulfides on Ti mesh via a hydrothermal process and these non-precious HC-NiMoS/Ti hybrids were explored as bifunctional catalysts for urea-based overall water splitting. They exhibited superior activity and stability with a cell voltage of 1.59 V for delivering 10 mA cm^−2^ in alkaline medium due to the highly exposed active sites, excellent charge transfer ability and good synergistic effects from multi-component reactions. 

**Figure 22:**
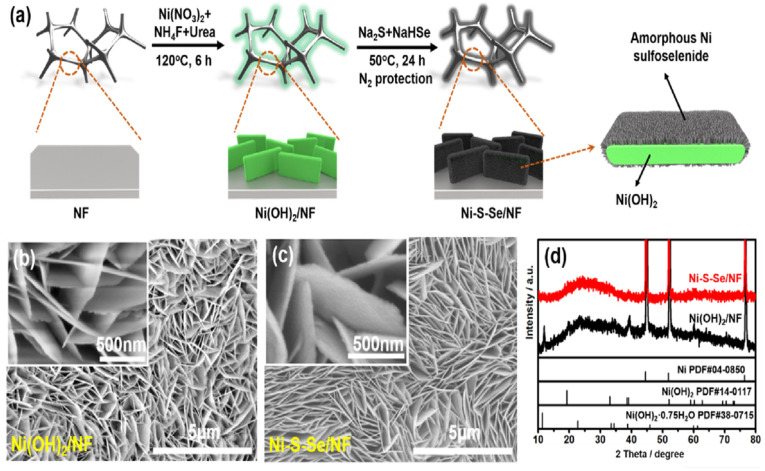
(**a**) Schematic illustration of the preparation of Ni-S-Se/NF, (**b**,**c**) SEM images and (**d**) XRD patterns of Ni(OH)_2_/NF and Ni-S-Se/NF, respectively. Reprinted with permission from Ref. [101].

**Figure 23:**
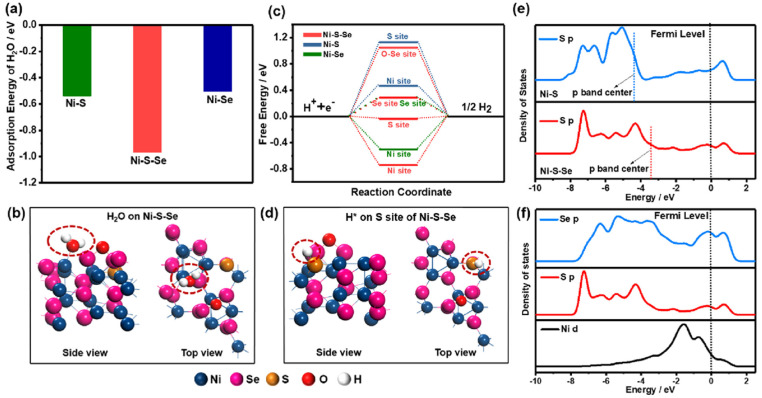
(**a**) Calculated water adsorption energy of Ni-Se, Ni-S and Ni-S-Se systems. (**b**) The calculated configuration of water adsorbed on the Ni-S-Se system (H_2_O in the circle). (**c**) Calculated adsorption free energy of H* on different sites of Ni-Se, Ni-S and Ni-S-Se systems. (**d**) The calculated configuration of H* adsorbed on the Ni-S-Se system (H* in the circle). (**e**) The DOS of sulfur’s p-orbital for Ni-S and Ni-S-Se systems; the p-band center is marked by dotted lines. (**f**) The DOS of Ni d-, S p- and Se p-orbitals of the Ni-S-Se system. Reprinted with permission from Ref. [101].

Recently, Maleki et al. [99] reported a highly active and stable bifunctional electrocatalyst of Ni-Mn-Se in NF and displayed overpotentials of 28 and 122 mV at 10 mA cm^−2^ for the HER and UOR, respectively. Additionally, it showed an overall urea-splitting voltage of 1.352 V at 10 mA cm^−2^. Bifunctional electrocatalytic activity of electrodeposited ternary NiMoSe on NF was published by Wang et al. [100] and only needed 1.39 V and 81 mV (vs. RHE) to deliver a current density of 10 mA cm^−2^ for the UOR and HER, respectively. Furthermore, to drive urea electrolysis, it only required 1.44 V to deliver a current density of 10 mA cm^−2^ and demonstrated good stability for urea electrolysis. Chen et al. [101] reported amorphous nickel sulfoselenide on the surface of Ni(OH)_2_ supported by NF using a hydrothermal technique and investigated its bifunctional activity for urea electrolysis. Illustration of the preparation of Ni-S-Se/NF, SEM images and its X-ray diffraction analysis are shown in Figure 22a–d, respectively. Theoretical studies manifested that Ni-S-Se/NF had higher water adsorption energy than Ni-Se/NF, and the S site in Ni-S-Se/NF presented the optimal hydrogen free energy for H_2_ formation. In addition, the Ni-S-Se/NF electrode also exhibited high activity for the UOR, and the active species were in situ-formed amorphous NiOOH (Figure 23a–f). The electrolyzer assembled by Ni sulfoselenide electrodes exhibited a low voltage of 1.47 at 10 mA cm^−2^ in 1 M KOH + 0.5 M urea, much lower than that of overall water splitting.

**Figure 24:**
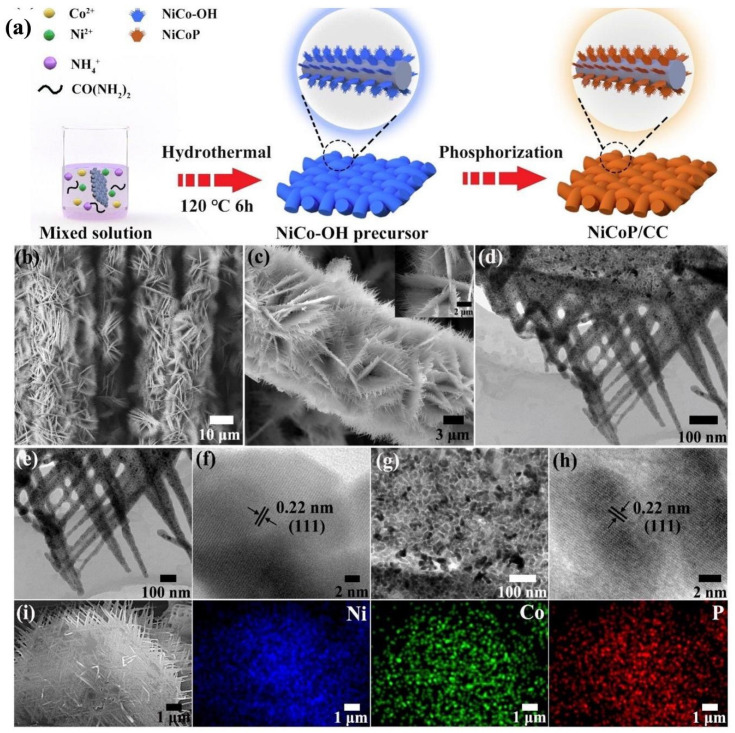
(**a**) Schematic illustration of the fabrication of NiCoP/CC; (**b**) low and (**c**) high-magnification SEM images; (**d**,**e**,**g**) TEM and (**f**,**h**) HRTEM images of the NiCoP/CC; and (**i**) SEM image and corresponding elemental mapping images of Ni, Co and P. Reprinted with permission from Ref. [103].

Xu et al. [102] fabricated unique core–shell in situ-grown Ni_12_P_5_ (core) ultrathin amorphous Ni phosphate (shell) nanorod arrays on NF (denoted as Ni_12_P_5_/Ni-Pi/NF) and studied their bifunctional electrocatalytic activity for both the UOR and HER. The attractive rod-like nanostructures combined with hierarchical 3D macroporous Ni collectors endowed the Ni_12_P_5_Ni-Pi/NF electrode with rich active centers and provided direct channels for the diffusion of produced gas products and the electrolyte ions into electrocatalysts. In addition, the Ni_12_P_5_/Ni-Pi/NF// Ni_12_P_5_/Ni-Pi/NF couple required just 1.532 V cell voltage to deliver 50 mA cm^−2^ in the two-electrode system and, for the current density of 500 mA cm^−2^, it required cell voltage as low as 1.662 V while simultaneously showing excellent durability during 6-h continuous electrolysis. Construction of self-supported leaf thorn-like nickel–cobalt bimetal phosphides as efficient bifunctional electrocatalysts for urea electrolysis was reported by Sha et al. [103]. Combined with the unique 3D architecture and the synergistic effect between Ni and Co, the as-obtained NiCoP/CC electrode delivered excellent HER and UOR electrocatalytic activities and the two-electrode urea electrolyzer needed a lower cell voltage of 1.42 V to deliver current density of 10 mA cm^−2^, less than that of overall water splitting. Their corresponding preparation method and SEM, HR-TEM and SAED images are explained in Figure 24a–i, respectively. A free-standing bifunctional electrocatalyst of P-NiFe@CF was synthesized by electroplating a Ni-Fe alloy onto carbon felt (CF), followed by phosphidation. The prepared P-NiFe@CF catalyst displayed excellent electrocatalytic activity for the UOR (demanding only 1.39 V (vs. RHE) to achieve 200 mA cm^−2^) and for the HER (with a low overpotential of 0.023 V (vs. RHE) at 10 mA cm^−2^). A urea electrolysis cell of P-NiFe@CF as both the free-standing anode and cathode reached a current density of 10 mA cm^−2^ at a cell potential of 1.37 V (vs. RHE), which is considerably lower than that of water electrolysis [104]. Yan et al. [105] reported the in situ growth of Ni_2_P/Fe_2_P/NF nanohybrids which displayed high activity for the HER at 115 mV and the UOR at 1.36 V with current density of 10 mA cm^−2^; a cell voltage of 1.47 V was needed to deliver the desired current density. 

### 4.3. Ni-Based MOFs and Nitrides

**Figure 25:**
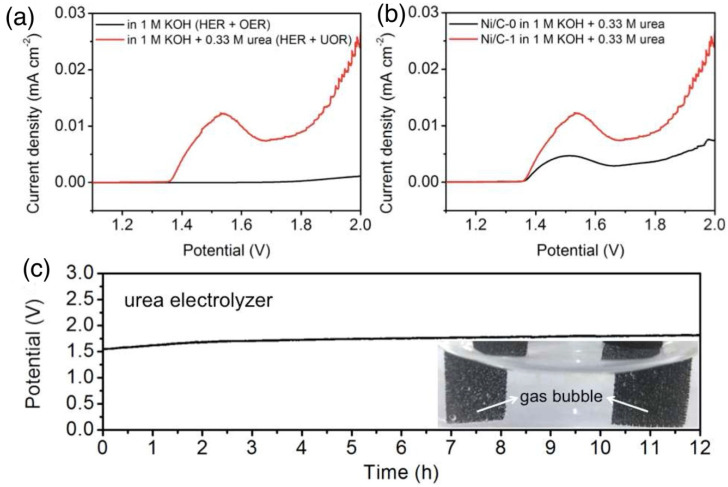
(**a**) LSV curves of an alkaline water electrolyzer and an alkaline urea electrolyzer using Ni/C-1 as a catalyst for both the HER and OER; (**b**) LSV curves of an alkaline urea electrolyzer using Ni/C-1 and Ni/C-0 catalysts; and (**c**) long-term durability tests of a urea electrolyzer. Inset: evolution of H_2_ and N_2_ gas. Reprinted with permission from Ref. [109].

MOFs are generally built by coupling metal clusters with organic ligands containing oxygen or nitrogen atoms, similar to a zeolite structure, and have potential applications in the booming fields of sensors, energy storage technologies and catalysis. Moreover, for fabricating highly porous materials with controlled morphologies, MOFs are considered to be promising due to their active centers in the well-defined carbon-based frameworks [106,107]. Numerous efforts have been made by researchers to develop Ni-based catalysts by using MOF precursors. Recently, Wang et al. [108] reported the preparation of Ni_2_P embedded Ni-MOF nanosheets (Ni_2_P@Ni–MOF/NF) through a direct phosphidation process, with the nanosheet directly used as a bifunctional electrocatalyst. They found that the overpotential was only 66 mV for the HER at 10 mA cm^−2^ and 1.41 V for the UOR at 100 mA cm^−2^. The electrolyzer constructed with a bifunctional electrode of Ni_2_P@Ni-MOF/NF delivered a current density of 100 mA cm^−2^ in 1 M NaOH with the presence of 0.33 M urea at 1.65 V, which was 0.26 V lower than water electrolysis. Wang et al. [109] synthesized highly porous pomegranate-like Ni/C using multivariate MOFs and demonstrated excellent HER activity, with an overpotential of 40 mV at 10 mA cm^−2^, and displayed UOR activity with onset potential of 1.33 V. Additionally, they assembled alkaline electrolyzers using Ni/C materials deposited on carbon cloth as catalysts for both the cathode and anode using 1 M KOH and 1 M KOH with 0.33 M urea as electrolytes. The cell exhibited higher activity with a smaller cell voltage of 1.6 V at the current density of 10 mA cm^−2^ in the presence of urea (Figure 25a), and the activity of Ni/C-1 was much higher than that of Ni/C-0 (Figure 25b). Finally, the stability of the urea electrolyzer was verified by chronopotentiometry at 10 mA cm^−2^ for 12 h (Figure 25c). The fabrication of 3D bimetallic Ni/Fe MOFs (MOF-Ni@MOF-Fe) was performed by Xu et al. [110] where, due to the influence of Fe, Ni_3_S_2_ was formed and produced MOF-Ni@MOF-Fe-S, which had superior UOR and HER activity of 1.347 V at and 0.145 V, respectively, at 10 mA cm^−2^ in 1.0 M KOH with 0.5 M urea. The assembled alkaline urea electrolyzer of MOF-Ni@MOF-Fe-S showed catalytic activity at a low cell voltage of 1.539 V at 10 mA cm^−2^ and excellent stability during 10 h of chronopotentiometry.

**Figure 26:**
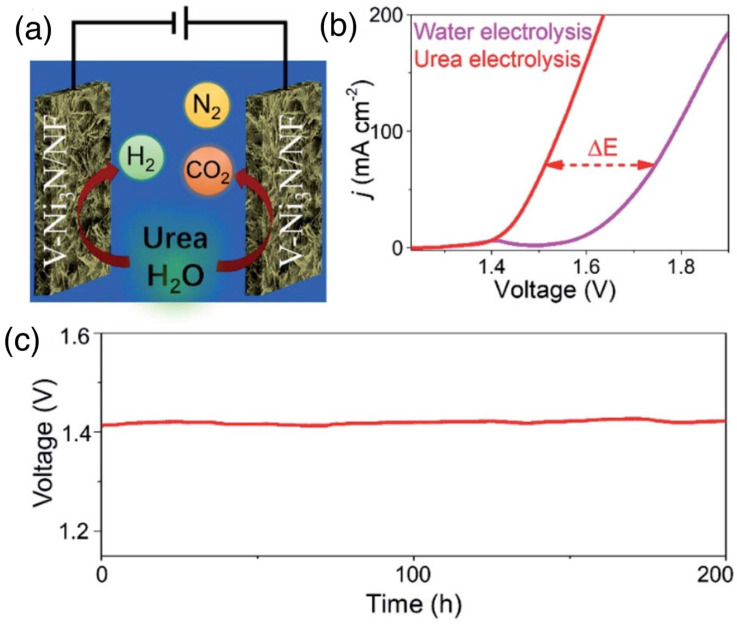
(**a**) Schematic illustration of the urea electrolyzer using V-NiN/NF HER and UOR. (**b**) Polarization curves of V-Ni_3_N/NF for urea and water electrolysis. (**c**) Long-term stability test performed at 10 mA cm^−2^. Reprinted with permission from Ref. [113].

Wang et al. [111] published a 3D composite of nickel nitride with reduced graphene oxide, Ni_3_N/rGO@NF, using various annealing temperatures; the electrode s-350 exhibited excellent UOR performance of 1.342 V and HER performance at overpotential of 124 mV at 10 mA cm^2^. Zhao et al. [112] reported the porous nickel nitride electrocatalyst Ni_3_N-350/NF, which was used as anode and cathode materials for water-urea electrolysis and displayed excellent catalytic activity and long-term stability for the HER and UOR. The assembled two-electrode electrolyzer (Ni_3_N-350/NF//Ni_3_N-350/NF) required lower voltage (1.51 V) to drive 100 mA cm^−2^ in water-urea electrolysis than for water electrolysis. Moreover, various electrodes of Ni_3_N-350/NF//Ni_3_N-350/NF, Ni_3_N-320/NF//Ni_3_N-320/NF, Ni_3_N-380/NF//Ni_3_N-380/NF and Pt/C//IrO_2_ needed voltages of 1.39 V, 1.37 V, 1.46 V and 1.51 V, respectively, at 20 mA cm^−2^, which confirmed the better performance of the Ni_3_N-350/NF material compared to the others. In addition, current measurement at an applied voltage of 1.51 V in water-urea electrolysis showed that Ni_3_N-350/NF//Ni_3_N-350/NF maintained stable current densities during 25 h of operation. The small fluctuations in current density near 20 h are caused by accumulation and release of bubbles on the electrode surface. Li et al. [113] developed porous V-doped Ni_3_N nanosheet arrays (V-Ni_3_N/NF) using a hydrothermal and subsequent nitridation process. Benefiting from abundant catalytically active sites and high electrical conductivity, they displayed low potentials of −83 mV and 1.361 V at 10 mA cm^−2^ for the HER and UOR, respectively. Furthermore, its two-electrode electrolyzer (Figure 26a) required low cell voltages of 1.416 V and 1.543 V to achieve 10 and 100 mA cm^−2^, respectively, whereas water electrolysis showed higher values of 1.596 V and 1.786 V at similar current densities (Figure 26b); excellent long-term stability upon 200 h of continuous electrolysis at 10 mA cm^−2^ was also demonstrated (Figure 26c). MOF-based nickel nitride was developed by Hu et al. [114], and the optimized material demonstrated 1.337 V at 10 mA cm^−2^ for the UOR and, at the same time, exhibited a low overpotential of 47 mV at 10 mA cm^−2^ for the HER. Table 3 showed the comparison of UOR and HER performance for bifunctional Ni-based electrocatalysts.

## 5. Summary and Outlook

Ni-based catalysts have been widely investigated since Botte’s pioneering work, reported in 2009, revealed the catalytic activity of Ni in alkaline solution among various metallic catalysts (Ni, Pt, Pt−Ir, Rh). Ni is one of the non-precious metals. Using cost-effective Ni-based electrocatalysts shows great potential for large-scale urea electrolysis. With the efforts of the past decade, the catalytic mechanisms of the UOR have been studied and proposed. Understanding the fundamental working principles is crucial for developing advanced UOR electrocatalysts. Nevertheless, applying Ni-based catalysts for practical urea electrolysis still faces several challenges. Here, future perspectives are discussed:

(1) Developing highly efficient UOR catalysts, in terms of boosted catalytic current, low overpotential and durable catalytic performance, is highly desirable. The majority of the prepared catalysts are nickel oxides and hydroxides at early stages, while nickel sulfides [115,116,117,118], selenides [62,63], phosphides [119,120] and nitrides [111] have shown appreciable UOR performance in recent years. In addition, Ni-based Prussian blue analogues [17] and perovskites [121] have also been revealed as promising candidates for the UOR. Thus, it is highly recommended to use the above-mentioned strategies to obtain diverse Ni−based catalysts. Scrutinizing these electrocatalysts by evaluating electrochemical performance and material/manufacturing cost is crucial for practical applications.
(2) Compared to the OER, the UOR has the potential to reduce the amount of energy consumed for hydrogen production significantly. However, practically, the oxidation potential of the UOR (>1.2 V vs. RHE) is generally much higher than its theoretical value (0.37 V vs. RHE) due to high overpotential. The difference in oxidation potential between the OER and UOR should theoretically be over 0.8 V but is actually less than 0.2 V because of the high overpotential required for the UOR. At the same time, practically, the OER also requires high overpotential due to its sluggish kinetics. Therefore, the development of efficient electrocatalysts to reduce the overpotential of the UOR is more important for effective hydrogen production through urea electrolysis.
(3) Electrochemical decomposition of urea involves multiple reaction steps and intermediates. Conducting in-depth studies on catalytic mechanisms is also important. So far, UOR mechanisms are only proposed for nickel oxide/hydroxide and Ni_2_Fe(CN)_6_-based catalysts. The roles of heteroatoms (such as S, Se, N, P and so on) and second/third metallic elements should be investigated. For defect engineering, the effects of structural defects (defect types and concentrations) on UOR catalytic activity should be discussed. Moreover, in situ characterizations are preferred to analyze the properties of catalysts while avoiding potential damage during post-treatment. DFT calculations can also provide valuable information for revealing the working principles of the prepared catalysts.
(4) For the HER, the main challenge for further development is the improvement of the activities and stabilities of HER electrocatalysts. Additionally, the most successful HER electrocatalysts should possess porous structures at the nanoscale, with large electrochemically active surface areas for fast charge transfer reaction on the surface, rather than the well-defined nanostructure morphologies.
(5) Recently, layered transition metal dichalcogenides MX_2_ were found to possess a hexagonal 2H structure and tetragonal 1T structure, with the stabilizing 1T structure being more significant due to its higher electric conductivity and electrocatalytic activity. On the other hand, due to the presence of abundant coordinatively unsaturated sites on the surface, amorphous materials have unique advantages toward the HER. Therefore, in future, attention should be given to the crystal structure and crystallinity of electrocatalysts for optimal HER electrocatalytic performance.
(6) Bifunctional electrocatalysts of Ni-based chalcogenides are inclined to undergo self-construction in the alkaline medium, and in situ techniques such as Raman spectroscopy, X-ray diffraction spectroscopy and X-ray absorption spectroscopy are therefore required for exploring the reaction intermediates, which will be more useful for better understanding the reaction mechanisms.
(7) Reducing manufacturing and material costs is also important for practical applications. Most of the Ni-based electrocatalysts are subjected to sophisticated preparation procedures, such as multi-step hydrothermal/solvothermal methods and/or high-temperature annealing conditions. Developing facile and energy saving methodologies for acquiring highly efficient electrocatalysts is highly recommended. For composite electrocatalysts, overall electrical conductivity can be effectively improved through the utilization of carbon supports such as graphene and carbon nanotubes. Nevertheless, using high-cost carbon-based materials would make the composite catalysts more economically unfavorable for practical applications.
(8) To further improve the economic and environmental significance of urea electrolysis, natural urine/urea-bearing wastewater should be utilized as an electrolyte for urea electrolyzers instead of chemical reagent-based electrolytes.
(9) Ultimately, to reduce costs and for convenient facilities, sunlight-powered photochemical urea electrolyzers should be developed for large-scale and commercial application of urea electrolysis.

## Figures and Tables

**Table 1:** Comparison of UOR performances of various Ni-based catalysts.

Catalysts	Onset Potential (V vs. RHE) at 10 mA/cm^2^	Current Density at 1.5 (V vs. RHE)	Electrolyte	Reference
Ni(OH)_2_ nanomeshes	1.35	~22 mA cm^−2^	1 M KOH + 0.33 M urea	[28]
Ni(OH)_2_ nanoflakes	~1.43	~50 mA cm^−2^	1 M KOH + 0.33 M urea	[30]
C@NiO	~1.32	~75 mA cm^–2^	1 M KOH + 0.33 M urea	[32]
Vertically aligned NiO nanosheets/NF	~1.38	~75 mA cm^–2^	1 M KOH + 0.33 M urea	[35]
NiS@Ni_3_S_2_/NiMoO_4_	~1.31	~148 mA cm^–2^	1 M KOH + 0.5 M urea	[42]
Ni_3_S_2_-Ni_3_P	~1.35	~150 mA cm^–2^	1 M KOH + 0.5 M urea	[43]
NiCo LDH-NO_3_	1.30	~58 mA cm^–2^	1 M KOH + 0.33 M urea	[46]
α−Ni(OH)_2_	1.40	58 mA cm^–2^	1 M KOH + 0.33 M urea	[47]
α−Ni(OH)_2_ with Ni vacancies	~1.41	~52 mA cm^–2^	1 M KOH + 0.33 M urea	[48]
NiCr hydroxide	~1.41	~42 mA cm^–2^	1 M KOH + 0.33 M urea	[51]
NiS_2_/SnS_2_	1.36	~75 mA cm^–2^	1 M KOH + 0.33 M urea	[57]
S-Ni(OH)_2_	1.32	~35 mA cm^−2^	1 M KOH + 0.33 M urea	[58]
Fe-Ni_3_S_2_	~1.37	~200 mA cm^–2^	1 M KOH + 0.33 M urea	[59]
Ni@N-doped CNT	~1.39	~36 mA cm^−2^	1 M KOH + 0.5 M urea	[64]

**Table 2:** Comparison of HER performance for Ni-based electrocatalysts.

Catalyst Material	Current Density	Overpotential for HER	Electrolyte	Reference
NiMo nanowire arrays	10 mA cm^−2^	17 mV	0.5 M H_2_SO_4_	[66]
Ni_3_N nanosheets	100 mA cm^−2^	100 mV	0.5 M H_2_SO_4_	[67]
Ni-Fe NP	10 mA cm^−2^	100 mV	1.0 M KOH	[69]
Ru_1_/D-NiFe LDH	10 mA cm^−2^	18 mV	1.0 M KOH	[70]
Ni-GF/VC	10 mA cm^−2^	128 mV	1.0 M KOH	[71]
h-NiS	10 mA cm^−2^	136 mV	1.0 M KOH	[73]
Ni-P–Pt/NF	10 mA cm^−2^	34 mV	1.0 M KOH	[74]
FeNi-MOF	10 mA cm^−2^	134 mV	0.1 M KOH	[79]
Ni_SA_-MoS_2_/CC	10 mA cm^−2^	98 mV	1.0 M KOH	[81]
	10 mA cm^−2^	110mV	0.5 M H_2_SO_4_	[81]
AC–Ni/NF	10 mA cm^−2^	30 mV	1.0 M KOH	[82]
Ni_3_N@CQDs	10 mA cm^−2^	69 mV	1.0 M KOH	[83]

**Table 3:** Comparison of UOR and HER performance for bifunctional Ni-based electrocatalysts.

Catalyst Material	Onset Potential or UOR	Current Density	Overpotential for HER	Current Density	Potential Required for Urea Electrolyzer	Reference
NiO/Ni_2_P	1.338 V	10 mA cm^−2^	137 mV dec^−1^	10 mA cm^−2^	1.457 V at 10 mA cm^−2^	[90]
NiFe-LDH/MWCNTs/NF	1.335 V	10 mA cm^−2^	98 mV dec^−1^	10 mA cm^−2^	1.507 V at 10 mA cm^−2^	[91]
NiFeCo LDH/NF	0.280 V (vs. SCE)	10 mA cm^−2^	108 mV dec^−1^	10 mA cm^−2^	1.49 V at 10 mA cm^−2^	[92]
(Ni_3_S_2_@NF)	0.36 V (vs. SCE)	100 mA cm^−2^	127 mV dec^−1^	10 mA cm^−2^	1.49 V at 20 mA cm^−2^	[96]
Ni_2_P/Ni_0.96_S	1.442 V	100 mA cm^−2^	239 mV dec^−1^	100 mA cm^−2^	1.453 V at 100 mA cm^−2^	[98]
HC-NiMoS/Ti)	1.38 V	60 mA cm^−2^	93.1 mV dec^−1^	10 mA cm^−2^	1.59 V at 10 mA cm^−2^	[99]
NiMoSe/NF)	1.39 V	10 mA cm^−2^	89 mV dec^−1^	10 mA cm^−2^	1.44 V at 10 mA cm^−2^	[101]
Ni-S-Se/NF	1.38 V	10 mA cm^−2^	98 mV dec^−1^	10 mA cm^−2^	1.47 V at 10 mA cm^−2^	[102]
Ni_12_P_5_/Ni-P/NF	1.337 V	100 mA cm^−2^	98.6 mV dec^−1^	10 mA cm^−2^	1.662 V at 500 mA cm^−2^	[103]
(NiCoP/CC)	1.30 V	10 mA cm^−2^	107 mV dec^−1^	10 mA cm^−2^	1.42 V at 10 mA cm^−2^	[104]
P-NiFe@CF	1.39 V	200 mA cm^−2^	23 mV dec^−1^	10 mA cm^−2^	1.37 V at 10 mA cm^−2^	[105]
Ni_2_P/Fe_2_P/NF)	1.36 V	10 mA cm^−2^	115 mV dec^−1^	10 mA cm^−2^	1.47 V at 10 mA cm^−2^	[106]
Ni/C	1.33 V	10 mA cm^−2^	40 mV dec^−1^	10 mA cm^−2^	1.6 V at 10 mA cm^−2^	[109]
MOF-Ni@MOF-Fe-S	1.347 V	10 mA cm^−2^	96 mV dec^−1^	10 mA cm^−2^	1.539 V at 10 mA cm^−2^	[110]
V–Ni_3_N/NF	1.361 V	10 mA cm^−2^	−83 mV dec^−1^	10 mA cm^−2^	1.416 V at 10 mA cm^−2^	[113]
Ni_3_N-350/NF	1.34 V	10 mA cm^−2^	128 mV dec^−1^	10 mA cm^−2^	1.51 V at 100 mA cm^−2^	[112]

## Data Availability

Not applicable.
